# Cooperative regulation of Zhx1 and hnRNPA1 drives the cardiac progenitor-specific transcriptional activation during cardiomyocyte differentiation

**DOI:** 10.1038/s41420-023-01548-1

**Published:** 2023-07-14

**Authors:** Yang Chen, Yukang Wu, Jianguo Li, Kai Chen, Wuchan Wang, Zihui Ye, Ke Feng, Yiwei Yang, Yanxin Xu, Jiuhong Kang, Xudong Guo

**Affiliations:** 1 Clinical and Translational Research Center of Shanghai First Maternity and Infant Hospital, Shanghai Key Laboratory of Maternal Fetal Medicine, Shanghai Key Laboratory of Signaling and Disease Research, Frontier Science Center for Stem Cell Research, National Stem Cell Translational Resource Center, School of Life Sciences and Technology, Tongji University, Shanghai, 200092 China; 2 Institute for Advanced Study, Tongji University, Shanghai, 200092 China

**Keywords:** Embryonic stem cells, Epigenetics

## Abstract

The zinc finger proteins (ZNFs) mediated transcriptional regulation is critical for cell fate transition. However, it is still unclear how the ZNFs realize their specific regulatory roles in the stage-specific determination of cardiomyocyte differentiation. Here, we reported that the zinc fingers and homeoboxes 1 (Zhx1) protein, transiently expressed during the cell fate transition from mesoderm to cardiac progenitors, was indispensable for the proper cardiomyocyte differentiation of mouse and human embryonic stem cells. Moreover, Zhx1 majorly promoted the specification of cardiac progenitors via interacting with hnRNPA1 and co-activated the transcription of a wide range of genes. In-depth mechanistic studies showed that Zhx1 was bound with hnRNPA1 by the amino acid residues (Thr111–His120) of the second Znf domain, thus participating in the formation of cardiac progenitors. Together, our study highlights the unrevealed interaction of Zhx1/hnRNPA1 for activating gene transcription during cardiac progenitor specification and also provides new evidence for the specificity of cell fate determination in cardiomyocyte differentiation.

## Introduction

Zinc finger proteins (ZNFs) are the most varied transcription factor family proteins, involved in a wide range of biological processes, including development, differentiation, metabolism, and autophagy [1, 2]. Numerous pieces of evidence have shown that many ZNFs are closely associated with cancer progression and neuro-related diseases such as Alzheimer’s disease, ischemic stroke, schizophrenia, epilepsy, and autism [3]. Despite all that, the proportion of well-known ZNFs is still quite low. Current studies have indicated that the ZNFs execute diverse biological functions dependent on their various types of zinc finger motifs endowing the intrinsic binding ability of DNA, RNA, and protein [4–7]. More importantly, the tandem combination of multiple zinc fingers significantly broadens the regulatory roles of ZNFs, enabling them to regulate gene expression through a variety of mechanisms in response to different cellular contexts or stimuli [8]. However, the current knowledge of the exact functions and various mechanisms of ZNFs is still limited.

The direct differentiation of pluripotent stem cells (PSCs) into cardiomyocytes in vitro is an important system for studying cardiac development and congenital heart diseases (CHDs) [9]. Although the PSC-derived cardiomyocytes (PSC-CMs) struggle with a lack of homogeneity and maturity and limited use in translational medicine, they are considered as the crucial cell source for treating adult heart diseases [10, 11]. Currently, the ZNFs have been reported to function in cardiac development and the occurrence of heart diseases. Deletion of the ZNF Casz1 or Zbtb20 led to cardiac insufficiency and ventricular septal defect [12, 13]. Knockout of Gata4 or Gata6 (containing zinc fingers) resulted in the downregulated expression of cardiac genes in early embryonic development [14, 15]. ZNF91 protects the heart by maintaining cardiac homeostasis under pressure overload and reversing isoproterenol-induced cardiac hypertrophy [16]. Both ZNF418 and ZNF307 were reported to counteract hypertensive overload-induced cardiac hypertrophy [17, 18]. Therefore, an in-depth understanding of the molecular regulation of specific ZNFs will shed light on the intricate regulatory network that governs cardiomyocyte differentiation and facilitates the development of stem cell translational medicine.

The zinc-fingers and homeoboxes (Zhx) family proteins belong to the ZNFs that possess both tandem zinc-finger domains and Homeobox domains [19]. Zhx proteins can bind with NF-YA to participate in gene transcription regulation [20]. The zinc-fingers and homeoboxes 1 (Zhx1), upregulated by the lncRNA-MALAT1/miR-199a axis, promoted glioma cell proliferation and progression [21]. Zhx2 inhibited the growth of hepatocellular cells as a tumor suppressor and was aberrantly expressed in multiple myeloma or Hodgkin lymphoma [22–24]. Zhx proteins also play an important role in the development and disease progression. Zhx1 formed heterodimers with Zhx3 to regulate the expression of podocyte-specific genes and renal function [25]. Zhx2 was specifically detected in the ventricular and subventricular zone of the cortex and correlated with the normal differentiation of cortical neural progenitors [26]. Sustainable downregulation of Zhx3 also reduced the expression of WT1, Lmx1b, and Pax2 genes which were crucial for focal glomerulopathy sclerosis [27]. However, the important and specific regulatory role of Zhx proteins in the fate decision of cardiomyocyte differentiation has not been revealed, which would significantly fill the deficiency of knowledge about the ZNF’s function in cardiac development.

Here, we found that Zhx1, transiently induced during the transition from mesoderm to cardiac progenitors (CPs), could positively regulate CP specification and cardiomyocyte differentiation in vitro. We also identified that hnRNPA1 was a cofactor of Zhx1 and co-regulated the expression of a series of genes during the CP specification. Mechanistic studies further revealed that Zhx1 was bound to hnRNPA1 via the amino acid residues (Thr111–His120) of the second Znf domain, thus achieving the specific regulation of Zhx1/hnRNPA1 interaction in cardiomyocyte differentiation.

## Results

### The ZNF Zhx1 is required for proper cardiomyocyte differentiation

**Fig. 1:**
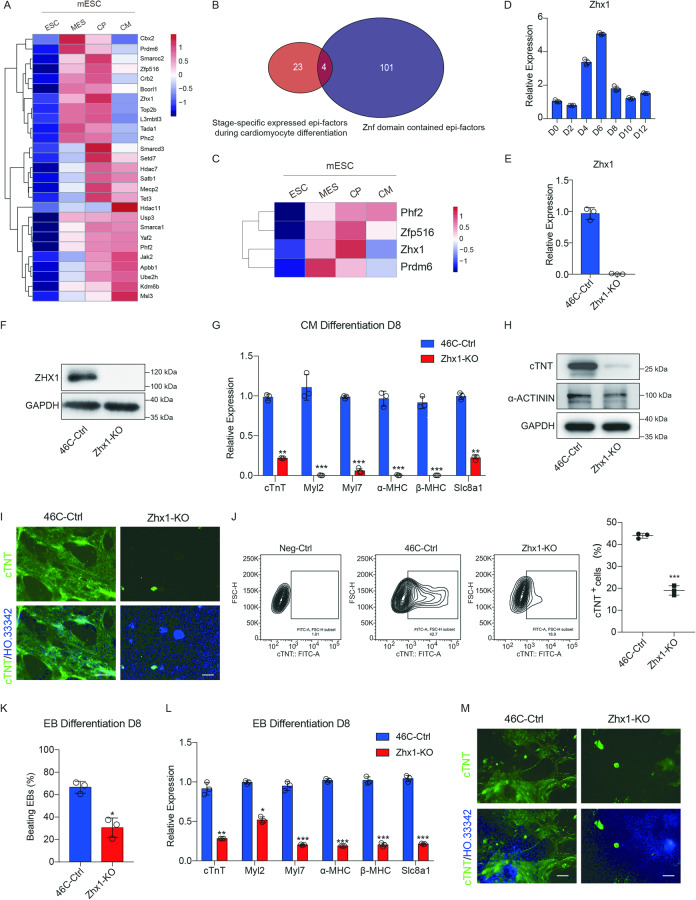
The ZNF Zhx1 is required for proper cardiac differentiation. **A** Heatmap shows the expression landscape of stage-specific upregulated epifactors in the indicated stages (embryonic stem cells (ESC), mesoderm (MES), cardiac progenitor (CP), and cardiomyocyte (CM)) of cardiomyocyte differentiation from mouse ESCs. **B** Cross-analysis for the epifactors contained the Znf domain. **C** Heatmap shows the expression of four epifactors contained the Znf domain during cardiomyocyte differentiation. **D** The expression of Zhx1 during the indicated days of cardiomyocyte differentiation. **E**, **F** Establishment of Zhx1 knockout mESCs (Zhx1-KO) via detecting mRNA (**E**) and protein level (**F**). **G** The expression of cardiomyocyte markers after Zhx1 knockout on day 8 of cardiomyocyte differentiation. **H** The western blot results for the expression of cTNT and α-Actinin on day 8 of cardiomyocyte differentiation after Zhx1 knockout. **I** The expression of cTNT protein after Zhx1 knockout during cardiomyocyte differentiation through immunofluorescent staining. **J** The percentage of cTNT^+^ cardiomyocytes after Zhx1 knockout. **K** The percentage of beating embryoid bodies (EBs) on day 8 of EB differentiation. **L** The expression of cardiomyocyte markers after Zhx1 knockout on day 8 of EB differentiation. **M** The expression of cTNT protein after Zhx1 knockout during the EB differentiation. Scale bar, 100 μm. Data are presented as the mean ± SEM (*n* = 3). The statistical significance is performed according to Student’s *t*-tests (unpaired two-tailed). *p < 0.05, **p < 0.01, and ***p < 0.001 versus 46C-Ctrl.

The differentiation of mouse embryonic stem cells (ESCs) into cardiomyocytes is a multistep process of cell fate transition. Numerous pieces of evidence have indicated that epigenetic factors (epifactors) are critical for stage-specific fate determination [9, 28, 29]. To investigate the key factors involved in the process of stage-specific cardiomyocyte differentiation, we screened the differentially expressed genes during mouse cardiomyocyte differentiation (embryonic stem cell stage, ESC; mesoderm stage, MES; CP stage, CP; cardiomyocyte stage, CM) and then cross-analyzed with the public epifactor database [30]. Twenty-seven stage-specific upregulated factors were identified (Fig. 1A). The zinc finger domain (Znf) endows the DNA recognition ability of epifactors, which is conducive to achieving the specificity of epigenetic regulation. We then identified 4 factors (Phf2, Zfp516, Zhx1, and Prdm6) with the Znf domains for further study (Fig. 1B, C). Prdm6 and Phf2, as the histone demethylases, were specifically up-regulated in the MES stage and gradually increased during cardiomyocyte differentiation. The ZNFs Zfp516 and Zhx1 were specifically expressed during the process of mesoderm to CPs, while Zfp516 had been reported to silence active genes and exit from pluripotency [31]. We then focused on the unrevealed function of Zhx1, which was indeed transiently upregulated during the transition from mesoderm to CPs (Fig. 1D and Supplementary Fig. S1).

We used the CRISPR/Cas9 gene editing technique to construct the Zhx1-knockout ESC cell line (Zhx1-KO) (Fig. 1E, F). We found that the deletion of Zhx1 did not affect the morphology and pluripotency-related gene expression of ESCs (Supplementary Fig. S2A–C). Whereas a significant decrease in the expression of cardiomyocyte markers was observed in the Zhx1-KO cells compared with control cells (46C-Ctrl) during cardiomyocyte differentiation (Fig. 1G). Similarly, the protein levels of cTNT and α-Actinin were significantly downregulated after Zhx1 knockout (Fig. 1H). Immunofluorescence and FACS assays also showed that loss of Zhx1 significantly decreased the percentage of cTNT^+^ cardiomyocytes (Fig. 1I, J, and Supplementary Videos I and II). Besides, our results showed that the deficiency of Zhx1 significantly increased the expression of marker genes of fibroblasts (Cola1, Col3a1, and Ckap4), endothelial cells (Pecam1 and Vecad), and smooth muscle cells (Desmin) (Supplementary Fig. S2D). To further determine the effect of Zhx1 deletion on spontaneous cardiomyocyte differentiation, we performed the embryoid body (EB) differentiation and also found that the deletion of Zhx1 could significantly decrease the number of beating EBs, the expression of cardiomyocyte genes and the percentage of cTNT^+^ cardiomyocytes (Fig. 1K–M). We further constructed two ZHX1-knockdown human embryonic cell lines (hESCs) (Supplementary Fig. S3A), and found that the knockdown of ZHX1 significantly reduced the differentiation efficiency of hESCs towards cardiomyocytes by inhibiting the expression level of cardiomyocyte markers, the protein level of CTNT and α-ACTININ, and the percentage of CTNT^+^ cardiomyocytes (Supplementary Fig. S3B–E and Supplementary Videos III and IV). In conclusion, the above findings suggested that the epifactor Zhx1 is indispensable for proper cardiomyocyte differentiation.

### Zhx1 specifically regulates the specification of CPs

**Fig. 2:**
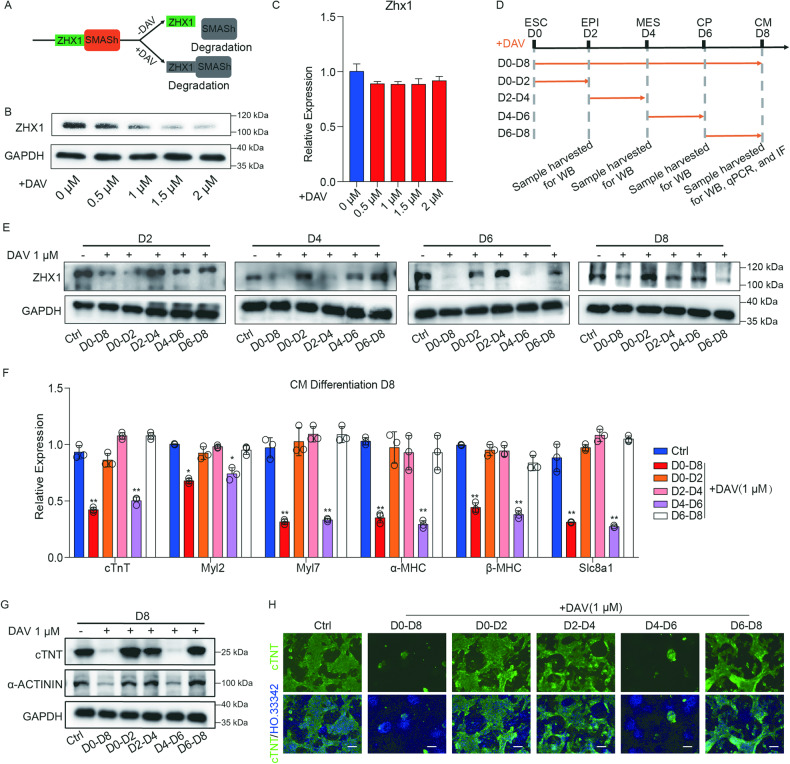
Zhx1 specifically regulates the specification of CPs. **A** The schematic diagram of SMASh-mediated ZHX1 degradation. **B**, **C** The protein (**B**) and mRNA (**C**) expression level of Zhx1 with the treatment of DAV in distinct concentrations (0, 0.5, 1, 1.5, and 2 μM). **D** A schematic diagram to represent the treatment timing and sample harvest during the SMASh-mediated ZHX1 knockdown. **E** The protein level of Zhx1 in the indicated days (D2, D4, D6, and D8) of cardiomyocyte differentiation after DAV treatment at different days (D0–D8, D0–D2, D2–D4, D4–D6, and D6–D8). **F** The expression of cardiomyocyte markers with the treatment of DAV (1 μM) at different days of cardiomyocyte differentiation. **G** The western blot results for the expression of cTNT and α-Actinin at day 8 of cardiomyocyte differentiation after DAV treatment (1 μM) at different days of cardiomyocyte differentiation. **H** The expression of cTNT protein with DAV treatment (1 μM) at different days of cardiomyocyte differentiation through immunofluorescent staining. Scale bar, 100 μm. Data are presented as the mean ± SEM (*n* = 3). The statistical significance is performed according to a two-way analysis of variance (ANOVA) followed by Bonferroni’s post hoc. *p < 0.05, **p < 0.01, and ***p < 0.001 versus Ctrl.

To explore the exact stage at which Zhx1 played an important role in regulating cardiomyocyte differentiation, we first analyzed the effects of Zhx1 knockout on the expression levels of specific genes in the epiblast (EPI), mesoderm cells (MES), and CP cells. Our results showed that depletion of Zhx1 did not affect the expression of genes related to early EPI cells and mesoderm cells but significantly influenced the expression level of markers related to CP cells (Supplementary Fig. S4A–D). The small molecule-assisted shut-off (SMASh) was a technique to reversibly modulate the expression level of target proteins, regardless of the nature, size, and subcellular location of target proteins [32, 33]. The SMASh would self-cleave and prevent the target protein from being degraded, while the self-cleave of SMASh was effectively blocked by adding the NS3 protease inhibitor like Danoprevir (DAV), leading to the degradation of the fusion target protein [32, 34]. We then constructed the ESC cell line (SMASh-Zhx1) capable of inducing the degradation of the Zhx1 protein (Fig. 2A). Our data showed that the expression of the Zhx1 protein, not mRNA, could be effectively reduced by adding 1 μM DAV (Fig. 2B, C). Then, we applied the cardiomyocyte differentiation with reduced expression of Zhx1 protein by adding 1 μM DAV at different stages (ESC-CM: D0–D8, ESC-EPI: D0–D2, EPI-MES: D2–D4, MES-CP: D4–D6, CP-CM: D6–D8) (Fig. 2D, E). Our results showed that only the knockdown of Zhx1 protein in the D4-D6 (the transition from MES to CP) could significantly inhibit cardiomyocyte differentiation by decreasing the mRNA level of cardiomyocyte markers, the protein level of cTNT and α-Actinin, and the percentage of cTNT^+^ cardiomyocytes, which were similar to the DAV treatment in the D0-D8 of cardiac differentiation (Fig. 2F–H). The above results suggested that Zhx1 mainly functions in cardiomyocyte differentiation by regulating the specification of CPs.

### Overexpression of Zhx1 promotes cardiomyocyte differentiation

**Fig. 3:**
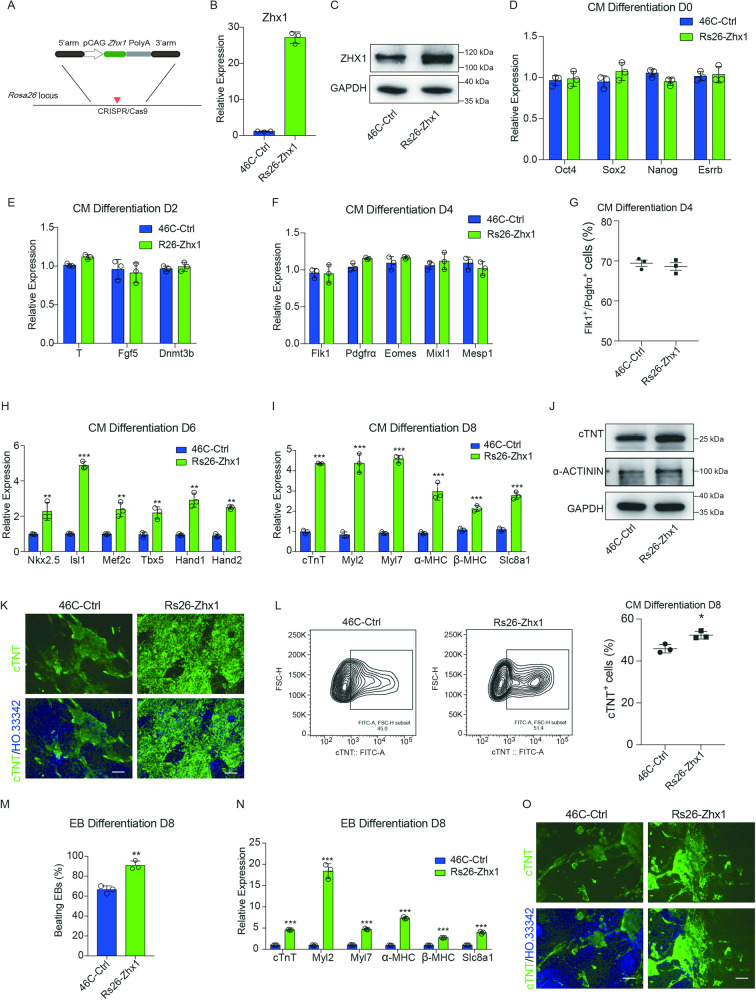
Overexpression of Zhx1 promotes cardiac differentiation. **A** The integrating strategy of the *Zhx1* gene in the *Rosa26* locus via CRISPR/Cas9 technology. **B**, **C** The mRNA (**B**) and protein levels (**C**) of Zhx1 overexpression. **D** The expression of pluripotent genes (Oct4, Sox2, Nanog, and Esrrb) after Zhx1 overexpression. **E** The expression of epiblast genes (T, Fgf5, and Dnmt3b) on day 2 of cardiomyocyte differentiation after Zhx1 overexpression. **F** The expression of mesoderm genes (Flk1, Pdgfrα, Eomes, Mixl1, and Mesp1) on day 4 of cardiomyocyte differentiation after Zhx1 overexpression. (**G**) The percentage of Flk1^+^/Pdgfrα^+^ cells on day 4 of cardiomyocyte differentiation after Zhx1 overexpression. **H**, **I** The expression of the CP genes (Nkx2.5, Isl1, Mef2c, Tbx5, Hand1, and Hand2) (**H**) and cardiomyocyte markers (cTnT, Myl2, Myl7, α-MHC, β-MHC, and Slc8a1) (**I**) after Zhx1 overexpression during cardiomyocyte differentiation. **J** The western blot results for the expression of cTNT and α-Actinin at day 8 of cardiomyocyte differentiation after Zhx1 overexpression. **K** The expression of cTNT protein after Zhx1 overexpression during cardiomyocyte differentiation through immunofluorescent staining. **L** The percentage of cTNT^+^ cardiomyocytes after Zhx1 overexpression. **M** The percentage of beating EBs on day 8 of EB differentiation after Zhx1 overexpression. **N** The expression of cardiomyocyte markers after Zhx1 overexpression on day 8 of EB differentiation. **O** The expression of cTNT protein after Zhx1 overexpression during the EB differentiation. Scale bar, 100 μm. Data are presented as the mean ± SEM (*n* = 3). The statistical significance is performed according to Student’s *t*-tests (unpaired two-tailed). *p < 0.05, **p < 0.01, and ***p < 0.001 versus 46C-Ctrl.

To further prove that Zhx1 is crucial for the cardiomyocyte differentiation process, we further inserted the full-length Zhx1 sequences driven by the CAG promoter into the *Rosa26* site to establish the Zhx1 overexpression cell line (Rs26-Zhx1, ~25-fold, Fig. 3A–C). Our finding showed that overexpression of Zhx1 did not affect the expression of genes related to ESCs, early EPI cells, and mesoderm cells but significantly influenced the expression level of marker genes related to CPs (Fig. 3D–H). Meanwhile, we found that overexpression of Zhx1 also increased the mRNA expression level of cardiac-specific genes, the protein level of cTNT and α-Actinin, and the percentage of cTnT^+^ cardiomyocytes compared with control cells (Fig. 3I–L and Supplementary Video V). We then performed the EB differentiation to confirm that overexpression of Zhx1 significantly improved the spontaneous differentiation ability of ESCs into cardiomyocytes by increasing the percentage of beating EBs, the expression levels of cardiac markers, and the number of cTNT^+^ cardiomyocytes (Fig. 3M–O). Taken together, our results indicated that Zhx1 is sufficient to promote cardiomyocyte differentiation.

### Genome-wide analysis of Zhx1/hnRNPA1 on gene activation

**Fig. 4:**
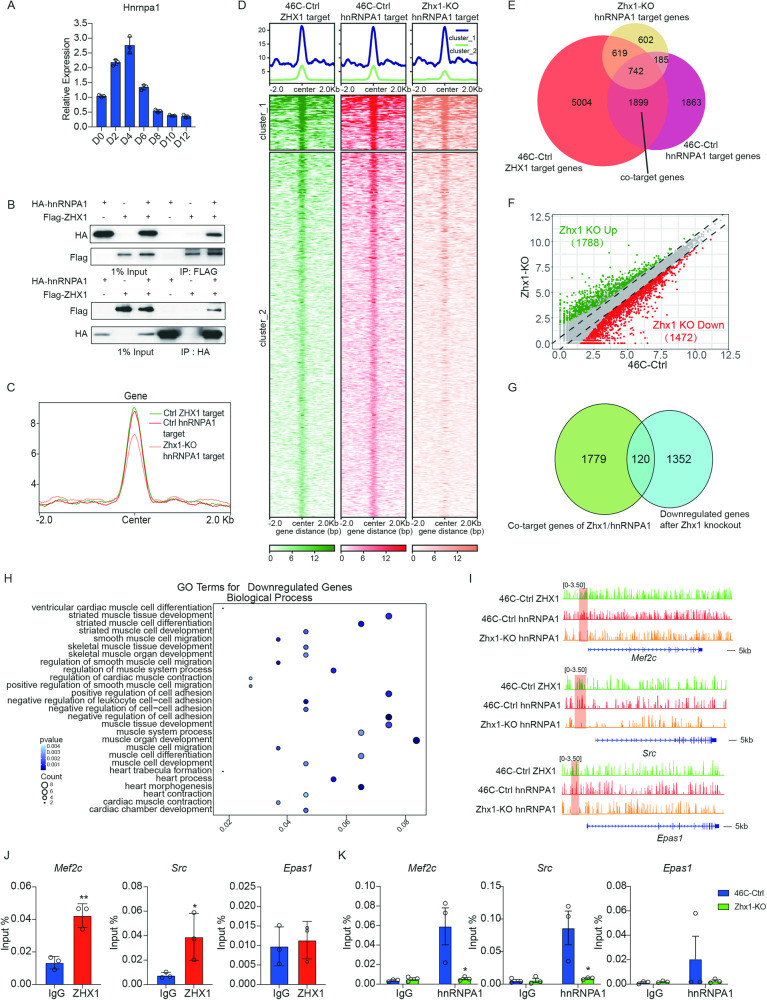
Genome-wide analysis of Zhx1/hnRNPA1 on gene activation. **A** The expression of Hnrnpa1 during the indicated days of cardiomyocyte differentiation. **B** Co-IP analysis for the interaction of ZHX1 and hnRNPA1. **C** The average profiles of ZHX1 and hnRNPA1 ChIP-seq at the genome-wide level in 46C-Ctrl cells and the hnRNPA1 ChIP-seq at the genome-wide level in Zhx1-KO cells. **D** Genome-wide heatmaps of the ZHX1 and hnRNPA1 enrichment of target genes in 46C-Ctrl cells, and the hnRNPA1 enrichment of target genes in Zhx1-KO cells. **E** Venn diagram shows the number of ZHX1/hnRNPA1 co-target genes in the CP stage. **F** RNA-seq scatterplot shows the upregulated (1788, green) and downregulated (1472, red) genes after Zhx1 knockout. **G** Cross-analysis for the co-target genes of ZHX1/hnRNPA1 and downregulated genes after Zhx1 knockout. **H** GO analysis for the co-target genes of ZHX1/hnRNPA1 which were downregulated after Zhx1 knockout. **I** Genome browser screenshots of ZHX1 and hnRNPA1 ChIP-seq on *Mef2c*, *Src*, and *Epas1* genes in 46C-Ctrl cells, and hnRNPA1 ChIP-seq on *Mef2c*, *Src*, and *Epas1* genes in Zhx1-KO cells. **J** ChIP analysis for the ZHX1 binding at the promoters of *Mef2c*, *Src*, and *Epas1* genes. **K** ChIP analysis for the hnRNPA1 binding at the promoters of *Mef2c*, *Src*, and *Epas1* genes after Zhx1 knockout. Data are presented as the mean ± SEM (*n* = 3). The statistical significance is performed according to Student’s *t*-tests (unpaired two-tailed). *p < 0.05, **p < 0.01, and ***p < 0.001 versus IgG or 46C-Ctrl.

To reveal the exact mechanism of Zhx1 in regulating the formation of CPs, we identified the proteins that potentially bound to Zhx1 in the CP stage by mass spectrometry (Table S4). Beyond the potential binding proteins, we chose the top 4 proteins (Hsd17b4, Krt16, Ywhaq, and hnRNPA1) for further analysis. Hsd17b4 is a peroxisome protein involved in peroxisome fatty acid metabolism, and lack of Hsd17b4 can lead to seizures, hearing and vision loss, and central nervous system symptoms [35]. Krt16 is a cytoskeleton protein and is mainly associated with congenital thick nail disease, psoriasis, and other skin diseases [36]. Ywhaq is a tyrosine 3-monooxygenase-activating protein associated with the development of liver and breast cancer [37]. Recent studies have shown that hnRNPA1 is critical for first heart field (FHF) and second heart field (SHF) formation during early heart development and mutations of hnRNPA1 often cause the CHDs in both humans and mice [38]. Therefore, we speculated that hnRNPA1 might be a cofactor of Zhx1 during the CP stage, which was also confirmed by Co-IP analysis (Fig. 4A, B).

To investigate whether the Zhx1–hnRNPA1 interaction was involved in gene transcription at the CP stage, we performed the genome-wide analysis of Zhx1 and hnRNPA1 binding with or without Zhx1. The results implied that the loss of Zhx1 significantly impeded the binding of hnRNPA1 on the genome (Fig. 4C, D). Venn diagram analysis for 2641 co-target genes overlapping with Zhx1 and hnRNPA1 in the 46C-Ctrl cells then revealed the binding of hnRNPA1 on 1899 genes was significantly impeded by Zhx1 depletion (Fig. 4E). Our results further showed that knockout of *Zhx1* gene led to upregulated (1788) and downregulated (1472) genes (Fig. 4F). Meanwhile, gene ontology (GO) and gene set enrichment analysis (GSEA) showed that these downregulated genes were mainly associated with biological processes such as heart development and muscle tissue formation (Supplementary Fig. S5A–C). Venn diagram analysis also showed that 120 genes co-targeted by Zhx1/hnRNPA1 interaction were downregulated after Zhx1 depletion (Fig. 4G). To further validate the cooperation between Zhx1 and hnRNPA1, we performed the GO analysis and found that these 120 co-targeted genes were mainly associated with cardiac muscle cell differentiation and heart morphogenesis (Fig. 4H). Though the Wnt or Smad2/3 pathway was critical for subsequent cardiac differentiation, the exact connection between Zhx1 with these pathways was not established (Supplementary Fig. S6A–D). The ChIP-seq data showed that the loss of Zhx1 resulted in the weakened binding of hnRNPA1 at the promoter regions of representative genes (*Mef2c*, *Src*, and *Epas1*) (Fig. 4I). We then validated the enrichments of Zhx1 and hnRNPA1 at the promoters of their potential co-targeted genes. Our results confirmed that Zhx1 was enriched at the promoters of *Mef2c* and *Src*, not *Epas1*, which were indeed bound by hnRNPA1 (Fig. 4J). The enrichment of hnRNPA1 at the promoters of *Mef2c* and *Src*, but not *Epas1*, was significantly impaired after Zhx1 knockout (Fig. 4K). To sum up, our results revealed a wide range of genes regulated by Zhx1-hnRNPA1 interaction and indicated that Zhx1 critically mediates the transcriptional activation of hnRNPA1 during the CP formation.

### Identification of the key amino acid residues of Zhx1 for binding hnRNPA1

**Fig. 5:**
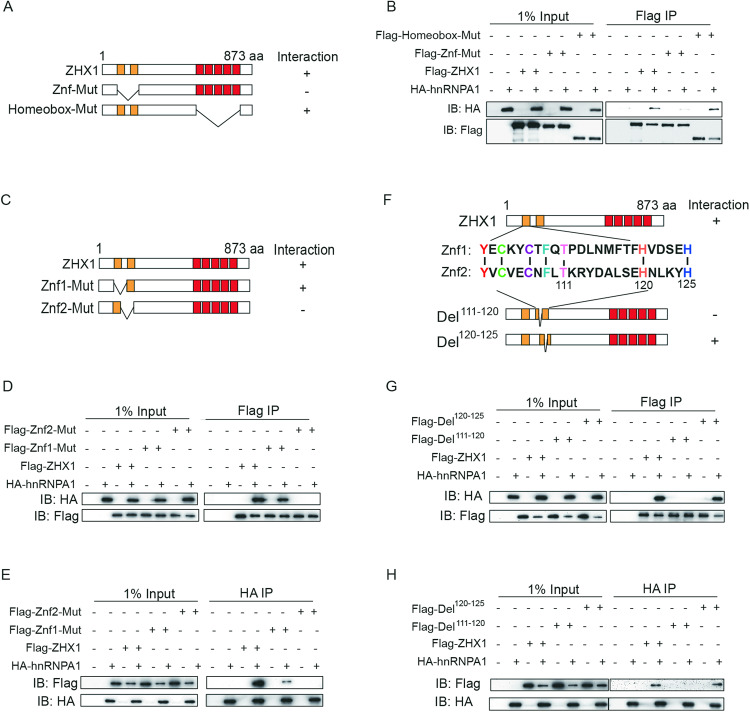
Identification of the key amino acid residues of Zhx1 for binding hnRNPA1. **A** The schematic of ZHX1 and ZHX1 mutants (Znf-mut and Homeobox-mut). **B** Co-IP analysis for the interaction of hnRNPA1 and ZHX1 or ZHX1 mutants (Znf-mut and Homeobox-mut). **C** The schematic of Znf mutants of ZHX1 (Znf1-mut and Znf2-mut). **D**, **E** Co-IP analysis for the interaction of hnRNPA1 and ZHX1 or Znf deletion mutants (Znf1-mut and Znf2-mut). **F** The alignment of Znf1 and Znf2 peptides. **G**, **H** Co-IP analysis for the interaction of hnRNPA1 and ZHX1 or ZHX1 deletion mutants (ZHX1-Del^120–125^ and ZHX1-Del^111–120^).

To identify the key domains for Zhx1/hnRNPA1 interaction, we performed the Co-IP of Flag-tagged Zhx1 mutants, including Znf domain deletion (Znf-Mut) and Homeobox domain deletion (Homeobox-Mut) and HA-tagged hnRNPA1 in HEK293FT cells. The results showed that the Znf domain of Zhx1 was responsible for binding hnRNPA1 (Fig. 5A, B). We further constructed the deletion mutants of Znf domain 1 (Znf1-Mut) and Znf domain 2 (Znf2-Mut). These results implied that Zhx1 was bound to hnRNPA1 through its Znf domain 2 (Fig. 5C–E). Since the conserved Znf domain, we aligned the Znf domain 1 and Znf domain 2 of mouse Zhx1 using ClustalX and focused on two relatively conserved motifs (Thr111–His120 and His120–His125) (Fig. 5F). Further Co-IP analysis showed that the Zhx1 mutant (Del^111-120^) lost its ability to bind with hnRNPA1 (Fig. 5G, H). Taken together, these results confirmed that the amino acid residues (Thr111-His120) in the Znf domain 2 of Zhx1 are essential for the binding of Zhx1 and hnRNPA1.

### Zhx1/hnRNPA1 interaction is responsible for the CP specification and cardiomyocyte differentiation

**Fig. 6:**
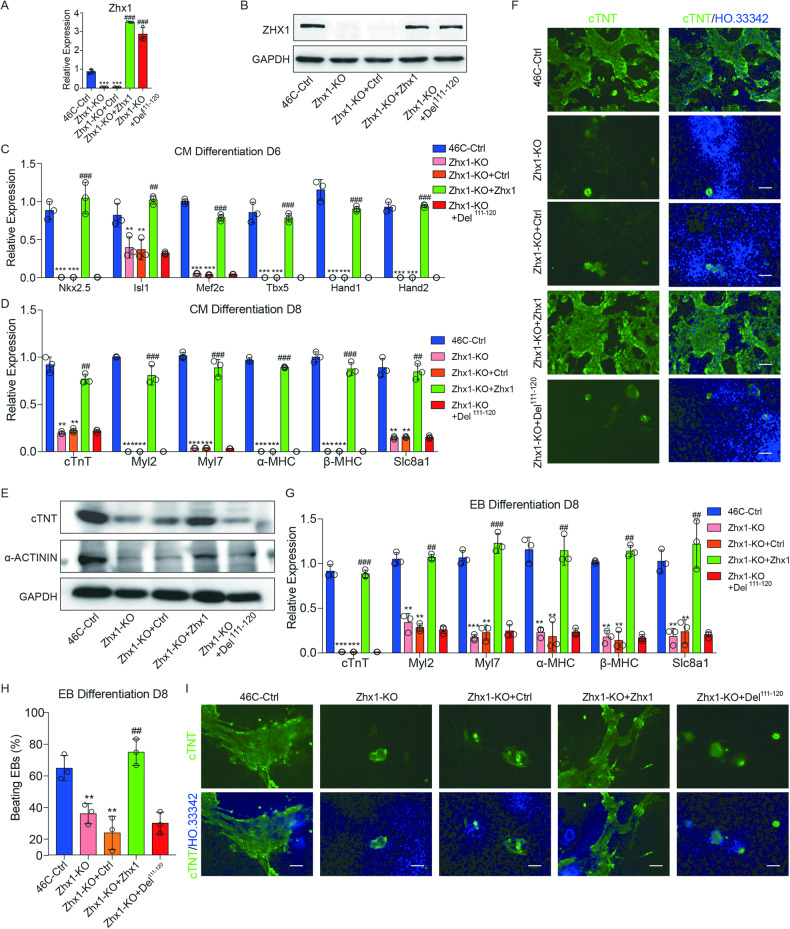
Zhx1/hnRNPA1 interaction is responsible for the CP specification and cardiomyocyte differentiation. **A**, **B** The mRNA (**A**) and protein levels (**B**) of overexpression of Zhx1 wild-type and deletion mutant (Del^111–120^) in Zhx1-KO cells. **C** The expression of the CP genes (Nkx2.5, Isl1, Mef2c, Tbx5, Hand1, and Hand2) after overexpressing wide-type Zhx1 or Zhx1 deletion mutant (Del^111–120^) in Zhx1-KO cells during cardiomyocyte differentiation. **D** The expression of cardiomyocyte markers after overexpressing wide-type Zhx1 or Zhx1 deletion mutant (Del^111–120^) in Zhx1-KO cells during cardiomyocyte differentiation. **E** The western blot results for the expression of cTNT and α-Actinin at day 8 of cardiomyocyte differentiation after overexpressing wide-type Zhx1 or Zhx1 deletion mutant (Del^111–120^) in Zhx1-KO cells. **F** The expression of cTNT protein after overexpressing wide-type Zhx1 or Zhx1 deletion mutant (Del^111–120^) in Zhx1-KO cells through immunofluorescent staining. **G** The expression of cardiomyocyte markers after overexpressing wide-type Zhx1 or Zhx1 deletion mutant (Del^111–120^) in Zhx1-KO cells during the EB differentiation. **H** The percentage of beating EBs on day 8 of EB differentiation after overexpressing wide-type Zhx1 or Zhx1 deletion mutant (Del^111–120^) in Zhx1-KO cells. **I** The expression of cTNT protein after overexpressing wide-type Zhx1 or Zhx1 deletion mutant (Del^111–120^) in Zhx1-KO cells during the EB differentiation. Scale bar, 100 μm. Data are presented as the mean ± SEM (*n* = 3). The statistical significance is performed according to a two-way analysis of variance (ANOVA) followed by Bonferroni’s post hoc. *p < 0.05, **p < 0.01, and ***p < 0.001 versus 46C-Ctrl; # *p* < 0.05, ## *p* < 0.01, and ### *p* < 0.001 versus Zhx1-KO+Ctrl.

To determine whether the Zhx1–hnRNPA1 interaction is required for cardiomyocyte differentiation, we performed the rescue experiments using wild-type Zhx1 and Zhx1 mutants (Del^111–120^). QPCR and western blot experiments confirmed that there was no significant difference in the expression of wild-type Zhx1 and Zhx1 mutants (Del^111–120^) in Zhx1-KO ESCs (Fig. 6A, B). As we expected, overexpression of wild-type Zhx1 could effectively rescue the gene expression of CPs, while the overexpression of Zhx1 mutants (Del^111–120^) failed (Fig. 6C). Subsequently, we further investigated the effects of Zhx1 expression on cardiomyocyte differentiation. Our results showed that the wild-type Zhx1 could significantly restore the downregulated cardiomyocyte marker gene expression, the decreased cTNT and α-Actinin protein levels, and the lower ratio of cTNT^+^ cardiomyocytes caused by Zhx1 deletion, whereas the Zhx1 mutant (Del^111–120^) could not recover the phenotypes of Zhx1 deletion (Fig. 6D–F). Consistently, the Zhx1 mutant (Del^111–120^) failed to rescue the phenotypes of decreased expression of the cardiomyocyte genes, decreased number of beating EBs, and low percentage of cTNT^+^ cardiomyocytes in the EB differentiation (Fig. 6G–I). Collectively, our data demonstrated that the Zhx1/hnRNPA1 interaction is indeed necessary for the CP specification and proper cardiomyocyte differentiation.

## Discussion

The differentiation of PSCs into cardiomyocytes sequentially goes through five stages: PSC, mesoderm, cardiac mesoderm, CP, and cardiomyocyte, and is an important system for studying stage-specific cardiac development and CHDs in vitro [9]. The orchestrated regulation of transcription factors and epigenetic modifiers is believed to determine the multi-stage cell fate decision during cardiomyocyte differentiation [39]. Our previous studies showed that lncRNA Cpmer relied on its RNA recognition specificity and post-transcriptionally regulated Eomes mRNA translation and mesoderm differentiation from PSCs, while lncRNA-1405 accurately scaffolded Eomes and epigenetic modifiers binding at the enhancer region of the *Mesp1* gene and drove cardiac mesoderm specification [40, 41]. SETD7 was also reported to bind BRG1/BAF60a and NKX2.5 to dictate mesoderm formation and the CP specification, respectively [42]. The cell fate specification of mesoderm to CPs, corresponding to the cardiac crescent originating from the lateral mesoderm during gastrulation in vivo, is the key step controlled by signals and regulatory factors, including ZNFs during cardiomyocyte differentiation. The BTB domain-containing ZNF CIBZ could repress the expression of the T and Mesp1 gene and cardiac mesoderm formation [43]. The myeloid zinc finger 1 (Mzf1), established as a hematopoietic transcription factor, blocked the formation of CPs by directly binding at the *Nkx2.5* enhancer [44]. Another ZNF factor CNBP/ZNF9 was bound with lncRNA Bvht and failed to activate key cardiac transcription factors in the CP stage [45]. However, the current knowledge of ZNFs involved in the specification of CPs from mesoderm is still limited. Our study uncovered that Zhx1 was transiently upregulated during the formation of CPs and significantly affected the ability of PSCs to differentiate into cardiomyocytes. The stage-specific inhibition of Zhx1 further confirmed that Zhx1 majorly functioned during the CP specification. Together, our study for the first time revealed that Zhx1 is an unrevealed factor that majorly controls the formation of CPs, which develops the comprehension of stage-specific differentiation of PSCs into cardiomyocytes.

Previous studies showed that Zhx1 was involved in carcinogenesis and glomerular disease by targeting specific genes. Zhx1 promoted the proliferation, migration, and invasion of glioblastoma cells and cholangiocarcinoma cells by activating the transcription of the EMT genes Snail2 and Twist1 or enhancing the Egr1 expression, respectively [46, 47]. While Zhx1 inhibited the apoptosis of glioblastoma cells by regulating the expression of Bcl2 and Bax and induced early proteinuria and primary glomerular disease by upregulating the Angptl4 expression [48, 49]. During the CP specification, we found that Zhx1 directly targeted many genes, which were significantly downregulated after Zhx1 knockout and closely associated with muscle cell differentiation, heart morphogenesis, and muscle organ development. Recent studies have indicated that Zhx1 not only formed homodimers but also formed heterodimers with Zhx2 dependent on an extensive region around the Homeodomain 1 (HD1) to regulate gene transcription in vivo and in vitro, formed heterodimers with Zhx3 which was a prerequisite for repressor activity [50, 51]. Besides, Zhx1 also achieved its regulation of a series of gene transcription by binding with the NF-Y subunit (NF-YA) [20], the co-repressor BS69 [52], or DNMT3B [53] to form a powerful transcriptional repressor complex and execute the transcription inhibition of broad genes. While the understanding of Zhx1 executes its transcriptional activation on a series of genes related to the CP specification is completely unrevealed. Our study revealed that Zhx1 interacted with hnRNPA1 and co-bound at the promoters of a large number of cardiac-related genes, such as *Mef2c* and *Src*, and activated their transcription. Therefore, our study first demonstrated that Zhx1 possesses the ability to directly target and activate a wide range of key genes in the CP specification.

The hnRNPA1 belongs to the hnRNP A/B subfamily and is involved in the regulation of alternative splicing of precursor mRNA, mRNA output and turnover, translation, miRNA processing, telomere length, and so on [54–57]. Previous studies have shown that PRMT catalyzed arginine methylation of hnRNPA1, which was able to regulate its binding activity and alternative splicing of target mRNAs in multiple cancers [58]. Recent studies have indicated that mutations of Hnrnpa1 caused the CHDs in both humans and mice, and Hnrnpa1 deletion resulted in the dysregulation of expression of cardiac transcription factors such as Isl1, Nkx2–5, and Tbx1 [38]. However, the mechanism by which hnRNPA1 was involved in cardiac defects and gene transcription implications has not been well elucidated. The hnRNPA1 could trigger transcriptional activation by binding at the promoter of the *ApoE* gene or smooth muscle cell genes like *Mef2c*, *Srf*, and *Myocd* [59, 60]. More importantly, hnRNPA1 was reported to unfold G-quadruplex DNA and functioned as a DNA destabilizing protein in the transcriptional regulation of human *KRAS* and *TRA2B* gene promoters [61, 62]. Our study identified that hnRNPA1 was the chaperone of Zhx1 and majorly bound with the amino acid residues (Thr111–His120) in the second Znf domain of Zhx1. The in-depth functional analysis confirmed that the binding region of Zhx1 and hnRNPA1 was responsible for executing the gene regulation during the CP specification. Together, our study indicated the unrevealed binding relationship of hnRNPA1 and Zhx1 might be endowed with the ability to actively unfold the DNA structure of gene promoters and facilitate the transcription activation of cardiac-related genes, which certainly suggested that the deletion or mutation of Hnrnpa1 might be an intrinsic factor for the hampered CP specification and cardiac defects.

In summary, our study indicated that Zhx1 interacted with hnRNPA1 and drove the specification of mesoderm to CPs, which facilitated the stage-specific transition of PSCs into cardiomyocytes. The in-depth mechanistic study demonstrated that the specific amino acid residues in the Znf domain were responsible for the binding of Zhx1 and hnRNPA1 and function execution. Thus, our study elucidated the new function and regulatory modes of Zhx1 in promoting the CP specification, developed our understanding of transcription regulation mediated by RNA-binding protein hnRNPA1, and also revealed an intrinsic interaction for the proper CP formation and cardiomyocyte differentiation.

## Materials and methods

### Cell culture and differentiation

Mouse ESCs (46C) were cultured on feeder cells with Dulbecco’s modified Eagle medium (DMEM) supplemented with 15% fetal bovine serum (FBS) (Gibco), 1× nonessential amino acid (Gibco), 1× GlutaMAX (Gibco), 1× sodium pyruvate (Gibco), 1 × 10^4^ units/mL leukemia inhibitory factor (LIF) (Millipore) and 55 μM β-mercaptoethanol (Gibco). The cells were passaged with 0.5 mL 0.25% trypsin (Gibco) at 37 °C for 1 min. Human ESCs (H9) were cultured on the Matrigel (BD) in the mTeSR1 medium (Stemcell). 293 T cells were cultured on the coated plate in the DMEM with 10% FBS (Gibco).

For EB differentiation, mESCs were cultured in a suspension medium (culture medium without LIF) to form EB spheres for 4 days in bacterial culture dishes (60 mm). Then, a single EB sphere was placed in gelatin-coated 24-well plates for adherent differentiation until day 8.

Cardiomyocyte differentiation was performed as previously described [9]. Briefly, mESCs were cultured in a serum-free medium for two passages and then trypsinized and suspended in a cardiomyocyte differentiation medium (CDM) containing 25 ng/mL l-ascorbic acid at a density of 5 × 10^4^ cells/mL. After 2 days, cells were subsequently trypsinized and resuspended in CDM containing 0.2 ng/mL hBMP4, 5 ng/mL hActivin A, 5 ng/mL VEGF, and 25 ng/mL l-ascorbic acid for 40 h. The EBs were trypsinized and attached to 12-well plates (pre-coated with gelatin) in a Stem Cell Pro-34 medium containing 5 ng/mL VEGF, 25 ng/mL FGF10, 4 ng/mL bFGF, and 25 ng/mL l-ascorbic acid at a density of 2.5 × 10^5^ cells/well for 4 days. (CDM: DMEM/DMEM-F12 (v/v = 1:1), 1× N2 supplement, 1× B27 supplement, 2 mM l-GlutaMax, and 4.5 × 10^−4^ M monothioglycerol). For human cardiomyocyte differentiation, hESCs were maintained on Matrigel-coated plates to achieve confluence, which was treated with CHIR99021 (8 μM) in the RPMI/B27 minus insulin medium for 48 h. Then, the medium was changed to RPMI/B27 minus insulin with IWP2 (5 μM) on day 3 for 48 h. Cells were maintained in the RPMI/B27 medium starting from day 7, with the medium changed every 3 days. All the cell lines were passed for the mycoplasma contamination test.

### Plasmid construction

Small guide RNAs (sgRNAs) targeting the *Rosa26* locus, the transcription start site, or the transcription stop site of the *Zhx1* gene were inserted into the pX330 vector, respectively. The sgRNAs were designed from http://crispr.mit.edu/. The Zhx1 overexpression donor contained the CAG-Flag-Zhx1-RBG PolyA sequences and bilateral homology arms of the *Rosa26* locus, which was cloned into the pLB vector (TIANGEN). The Zhx1-SMASh donor contained the SMASh domain, the PGK-BSD-RBG PolyA segment, and the bilateral homology arms nearby the stop site of the *Zhx1* gene, which was also cloned into the pLB vector. The shRNAs targeted ZHX1 were cloned into the pLKO.1 vector. The primer sequences are listed in Table S1.

### Cell line establishment

To generate the *Zhx1* knock-out mESCs, 5 μg pX330-sgRNAs respectively targeting the transcription start site and transcription stop site of the *Zhx1* gene were mixed with the P3 Primary cell solution (Lonza) and synchronously electroplated into wild-type 46 C mESCs using the CG-104 program of 4D Nucleofector system according to the manufacturer’s instructions. To generate the Zhx1 constitutive overexpression mESCs, 5 μg pX330-sgRNA targeting the *Rosa26* locus and 10 μg Zhx1 overexpression donor were mixed with the P3 Primary cell solution and co-electroporated into 46 C mESCs. To generate the Zhx1-SMASh knock-in mESCs, 5 μg pX330-sgRNA targeting the transcription stop site of the *Zhx1* gene and 10 μg Zhx1-SMASh donor were mixed with the P3 Primary cell solution and co-electroporated into 46 C mESCs. After electroporation and drug selection, PCR was used to identify the proper cell lines. To generate the shZHX1 hESCs, the H9 cells were infected by the shZHX1 lentivirus, which was packaged in 293 T cells and concentrated with Lenti-Concentin Virus Precipitation Solution (ExCell).

### Fluorescence activating cell sorter (FACS)

The EBs produced on day 4 of cardiomyocyte differentiation were dissociated with 0.125% trypsin (Gibco) and washed with 1× PBS. The dissociated cells (2.5 × 10^5^) were incubated with 200 μL PBS containing 1 μL of CD140a (Pdgfrα)-APC antibody (130-109-784, Miltenyi Biotec.) and 1 μL of CD309 (Flk1)-PE antibody (130-102-559, Miltenyi Biotec.) at room temperature for 30 min. For the analysis of cardiomyocyte differentiation efficiency, the cells on day 8 of cardiomyocyte differentiation were dissociated with collagen I and washed with 1× PBS, which were sequentially fixed and permeabilized with fixation and permeabilization solution (BD Biosciences) for 20 min, incubated with the cTNT antibody for 1 h and fluorescent secondary antibody for 40 min. Cells were analyzed with BD FACSVerse, and the data were analyzed with FlowJo software.

### Mass spectrometry (MS)

The CPs were lysed with IP lysis buffer on ice for 30 min. The IP assay was performed using magnetic beads incubated with an anti-Zhx1 antibody. Then, the Zhx1 binding samples were analyzed by LC–MS as described previously [63].

### Quantitative RT-PCR (qPCR)

Total RNA was extracted from cells with TRIzol reagent (Invitrogen), and 500 ng RNA was reverse-transcribed with PrimeScript RT reagent kit (TaKaRa). qPCR was performed with SYBR green reagent (Bio-Rad) in the MX3000 qPCR system (Agilent). The primers used for qPCR are listed in Table S2.

### Immunostaining

The cells were fixed in 4% paraformaldehyde for 15 min at room temperature, washed with 1× PBS, and then permeated with 0.2% Triton X-100 for 8 min. Staining was performed with primary antibodies overnight, secondary fluorescent antibodies for 2 h, and nuclear staining (Hoechst 33342) for 20 min. Images were obtained with a Nikon A1R confocal microscope.

### Western blotting

The cells were dissolved in 1× SDS solution for 30 min on ice. Protein lysates were separated by SDS-PAGE and transferred to PVDF membranes, which were incubated with primary antibodies overnight at 4 °C and secondary antibodies for 2 h at 4 °C. The primary antibodies used in western blot experiments were as follows: anti-Zhx1 (Novus, NB600-244), anti-Flag (CST, 14793 s), anti-HA (Abcam, ab9110), anti-α-Actinin (Sigma, A7811), anti-p-Smad2/3 (Bioworld, BS1838), anti-Smad2/3 (CST, 5678 s), anti-Active β-Catenin (Millipore, 05665), anti-Total β-Catenin (Abcam, ab16051), and anti-GAPDH (Bioworld, AP0063) (Table S3). All the full-length western blots were included in the Supplementary Data.

### Co-immunoprecipitation (Co-IP)

The Co-IP was performed as previously described [64]. The following affinity gel was used in the Co-IP: Ezview Red Anti-FLAG M2 affinity gel (Sigma, F2426) and Ezview Red Anti-HA affinity gel (Sigma, E6779). All the full-length western blots were included in the Supplementary Data.

### Chromatin immunoprecipitation (ChIP)

The ChIP was performed as previously described [40]. The following antibodies were used in the ChIP assay: anti-Zhx1 (Novus, NB600-244) and anti-hnRNPA1 (Novus, NB100-672).

### Sequencing data analysis

For RNA-seq analysis, cutadapt (V1.18) was used to remove adapter sequences, low-quality bases, and reads shorter than 50 bases. The trimmed clean data were mapped to the mouse reference genome (GENCODE GRCm38, release M19) using HISAT2 (V2.1.0) with the parameters “--rna-strandness RF --dta-cufflinks --no-discordant”. After that, gene expression levels were quantified as FPKM by stringtie (V1.3.4d) with the parameter “-e --rf”. Genes with FPKM < 0.1 in all samples were filtered, and FPKM values of replicates were averaged. For ChIP-seq analysis, the data were first filtered using cutadapt (V1.18) to remove adapter sequences, low-quality bases and reads shorter than 50 bases. After quality control, the clean data were mapped to the mouse reference genome (GENCODE GRCm38, release M19) by bowtie2 (V2.3.5.1) with the following parameters: -N 1 -X 2000 -q -t -L 25 --no-mixed --no-discordant. The unmapped reads, nonuniquely mapped reads, and PCR duplicates were removed. Next, the read counts were normalized to RPKM (reads per kilobase per million) in 100-bp bins for downstream analysis. The Zhx1 and hnRNPA1 ChIP-seq signals were visualized using the Integrative Genomics Viewer (IGV) browser (V2.4.10) and deepTools suite (V3.3.0).

### Statistical analyses

All the statistical data were presented as the mean ± S.E.M. of three independent experiments. The data in this study were analyzed with Student’s *t*-tests (unpaired two-tailed) or two-way analysis of variance (ANOVA) followed by Bonferroni’s post hoc. *^,#^*P* < 0.05, **^,##^ *P* < 0.01, and ***^,###^ *P* < 0.001.

## Supplementary information

Supplementary data

Original Data File

Co-author agreement

Suppmentary Video I

Suppmentary Video II

Suppmentary Video III

Suppmentary Video IV

Suppmentary Video V

## Data Availability

The RNA-seq and ChIP-seq datasets produced in this study are available in the Gene Expression Omnibus (GEO) database (accession number: GSE234336).
