# Supplementary material for: Cooperative regulation of Zhx1 and hnRNPA1 drives the cardiac progenitor-specific transcriptional activation during cardiomyocyte differentiation
Source: Cell Death Discov. 2023 Jul 14;9:244. doi: 10.1038/s41420-023-01548-1 (PMC10349095; doi:10.1038/s41420-023-01548-1)
Supplement: Supplementary file 1 — Supplementary data [file 41420_2023_1548_MOESM1_ESM.docx]

**Supplementary data**

**Inventory of Supplemental Information**

**Supplemental Figures and Figure Legends**

Supplementary Fig. S1, related to Figure 1

Supplementary Fig. S2, related to Figure 1

Supplementary Fig. S3, related to Figure 1

Supplementary Fig. S4, related to Figure 2

Supplementary Fig. S5, related to Figure 4

Supplementary Fig. S6, related to Figure 4

**Supplemental Tables**

Table S1, Primers used for vector construction.

Table S2, Primers used for RT-qPCR and ChIP-qPCR assays.

Table S3, Antibodies used in this study.

Table S4, The proteins interacted with ZHX1 identified by mass spectrometry assay.

**Supplemental Figures and Figure legends**


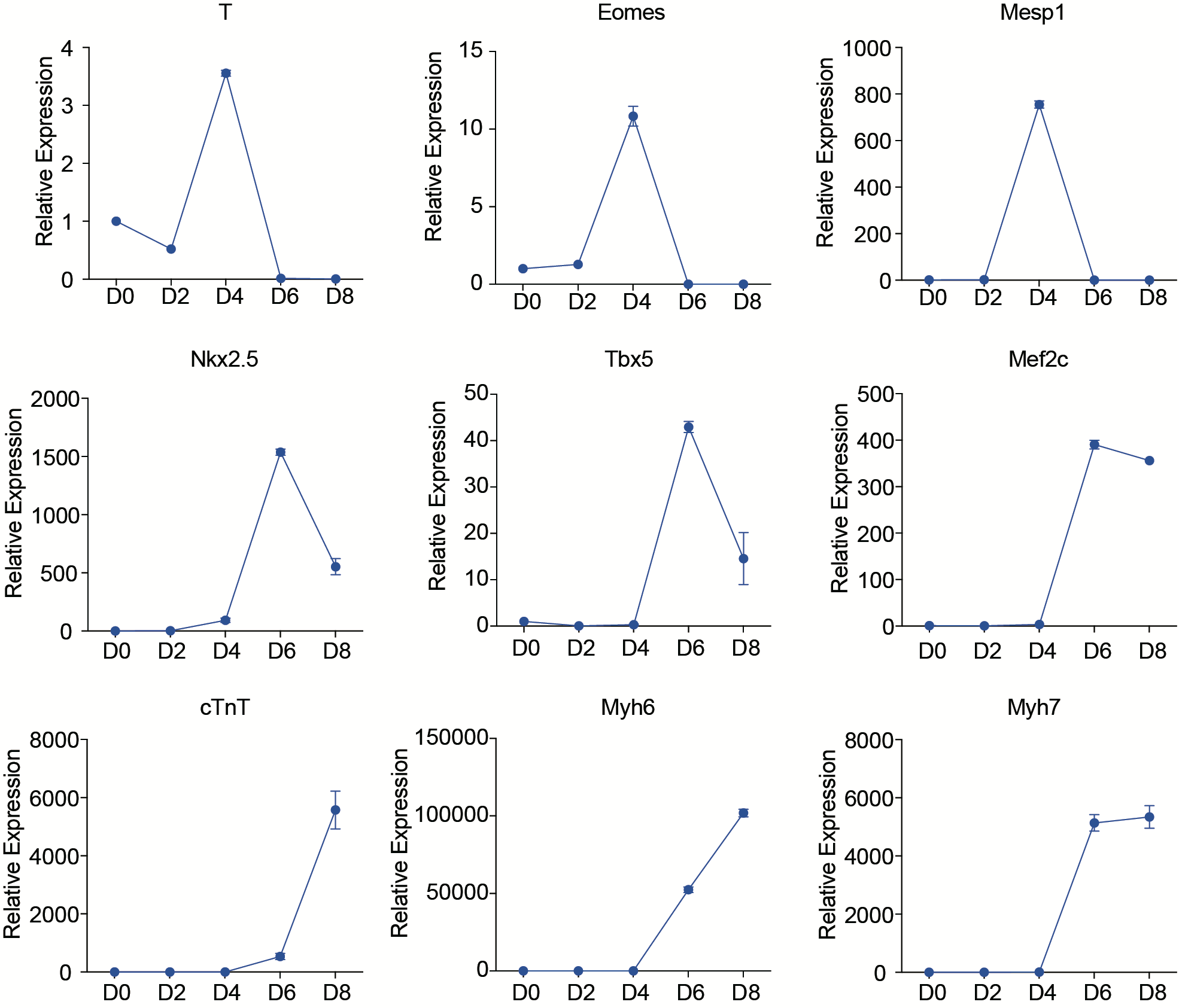


**Supplementary Fig. S1. The expression level of stage-specific markers during cardiomyocyte differentiation.** The expression levels of stage-specific markers such as T and Eomes (mesoderm markers), Mesp1 (cardiac mesoderm marker), Nkx2.5, Tbx5, and Mef2c (cardiac progenitor markers), Myh6, Myh7, and cTnT (cardiomyocyte markers) during cardiomyocyte differentiation. Data are presented as the mean ± SEM (n = 3).

**
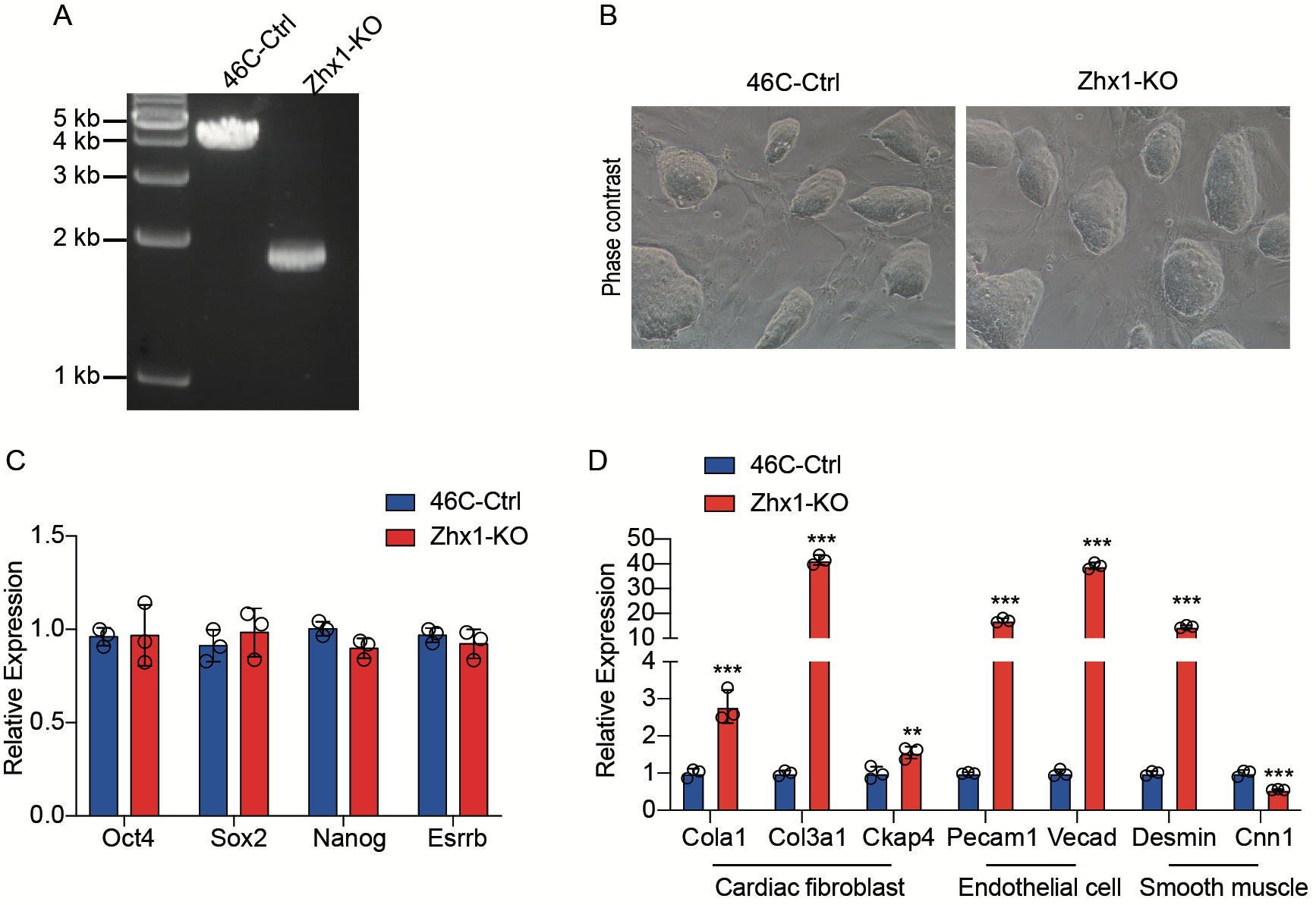
**

**Supplementary Fig. S2. Zhx1 deletion does not affect ESC morphology and pluripotency gene expression.** (A) PCR analysis for the *Zhx1* gene deletion. (B) The representative images of Zhx1-KO mESCs. (C) The expression of pluripotent genes (Oct4, Sox2, Nanog, and Esrrb) after Zhx1 knockout. (D) The qPCR detection for marker genes of fibroblasts (Cola1, Col3a1, and Ckap4), endothelial cells (Pecam1 and Vecad), and smooth muscle cells (Desmin and Cnn1) after Zhx1 knockout. Data are presented as the mean ± SEM (n = 3). The statistical signiﬁcance is performed according to Student’s t-tests (unpaired two-tailed). *p < 0.05, **p < 0.01, and ***p < 0.001 versus 46C-Ctrl.


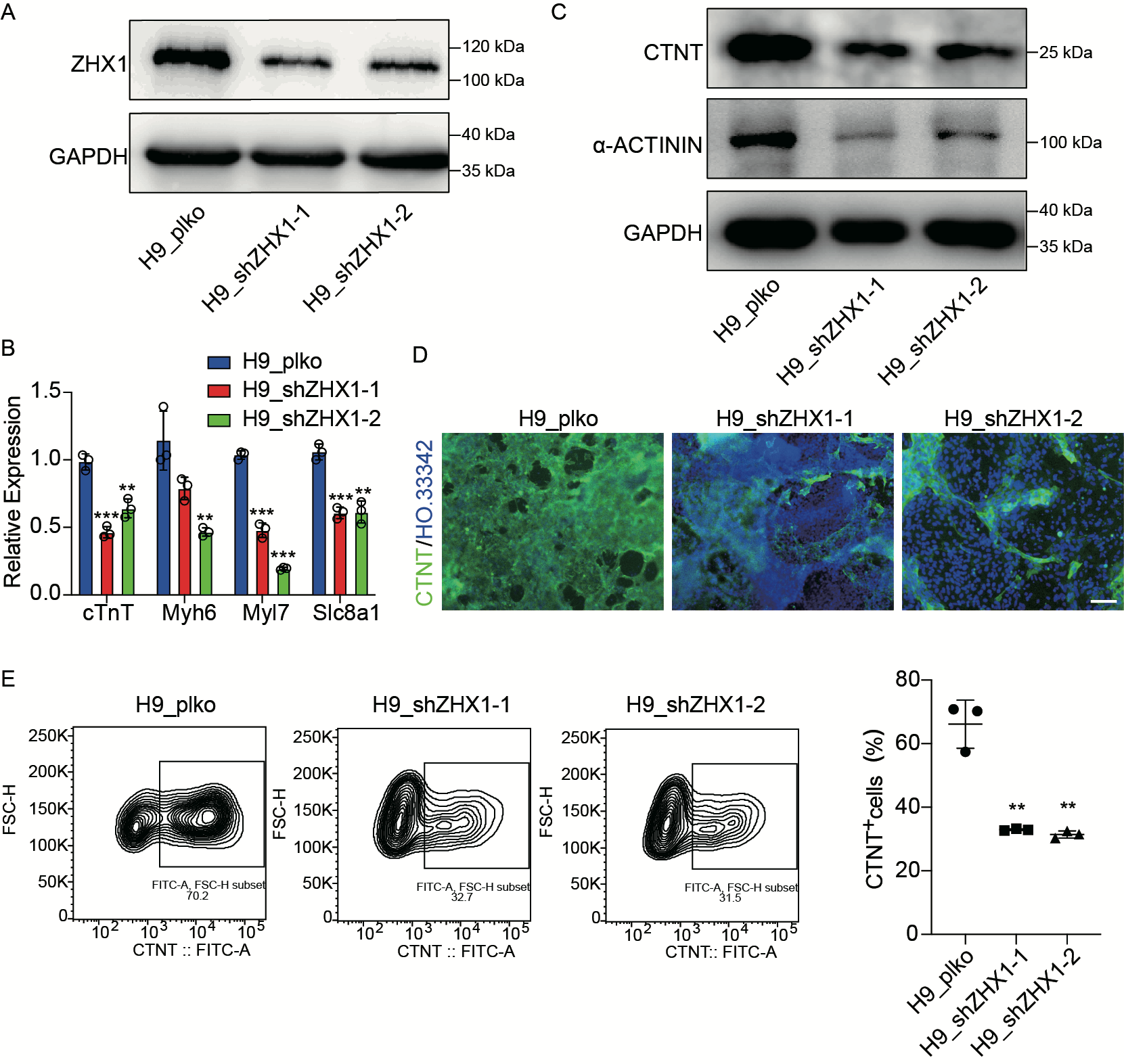


**Supplementary Fig. S3. Knockdown of ZHX1 similarly blocks the human cardiomyocyte differentiation.** (A) The knockdown efficiency of shRNA targeting ZHX1 in human ESCs. (B) The mRNA expression levels of cardiomyocyte marker genes after ZHX1 knockdown during human cardiomyocyte differentiation. (C) The western blot results for the expression of CTNT and α-ACTININ at day 10 of human cardiomyocyte differentiation after knocking ZHX1 down. (D) The [immunofluorescent staining](http://www.baidu.com/link?url=o_2P9kjOO6Dq3ov-3MzCJYlbOEZu0tQQovnCwrUsT-XyJL31qqI95O0Anojt-_joMmL6Pqjq8MzlEIwRq-quJvibh1EA7oNmSPXkFIWwSSbC8UHpSG3_1AMnzoqFds6M) of CTNT^+^ cardiomyocytes after ZHX1 knockdown. Scale bar, 100 μm. (E) The FACS analysis for the percentage of CTNT^+^ cardiomyocytes after ZHX1 knockdown during human cardiomyocyte differentiation. Data are presented as the mean ± SEM (n = 3). The statistical signiﬁcance is performed according to Student’s t-tests (unpaired two-tailed). *p < 0.05, **p < 0.01, and ***p < 0.001 versus H9_plko.


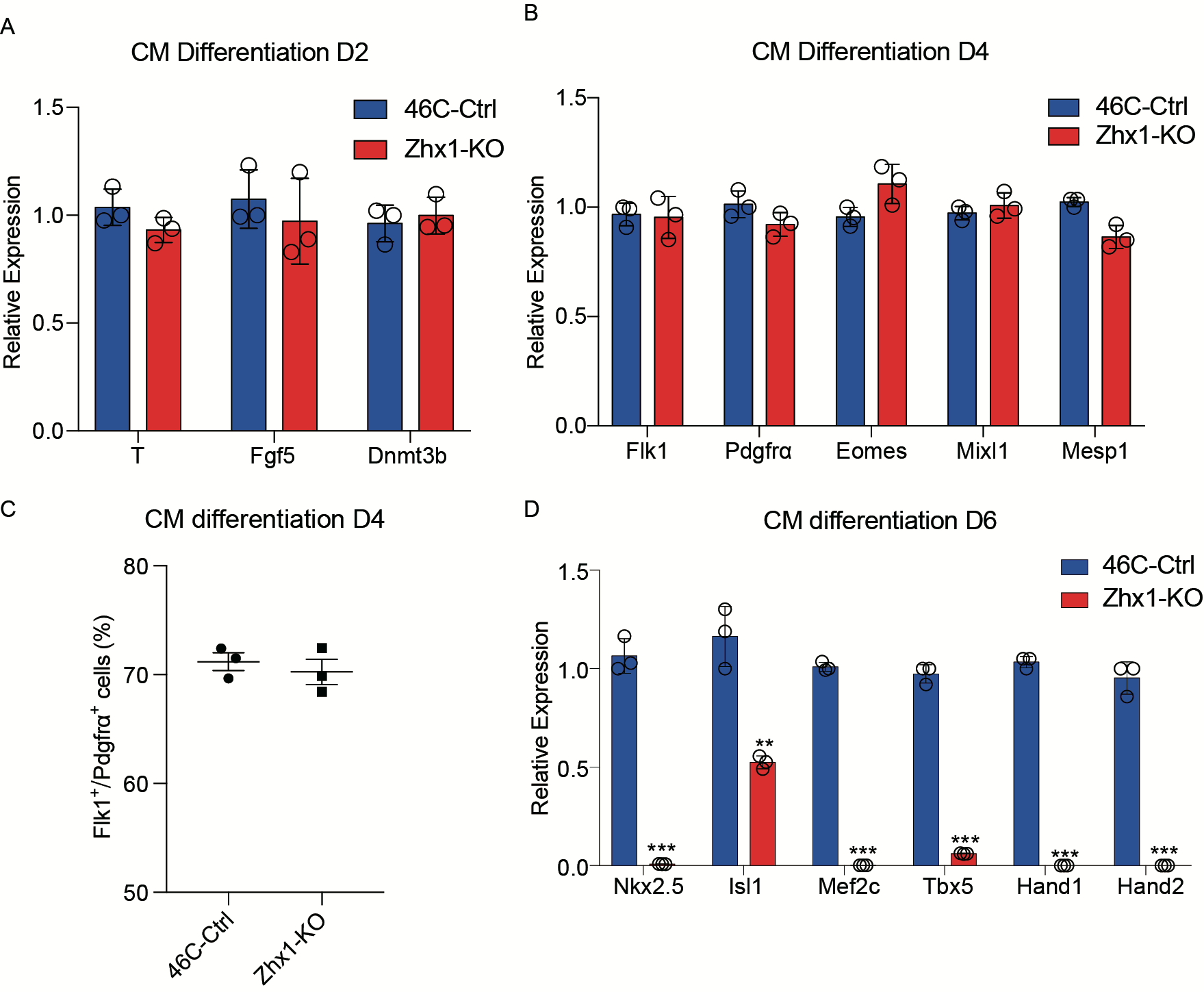


**Supplementary Fig. S4. Knockout of Zhx1 significantly inhibited the expression level of cardiac progenitor genes, not the epiblast and mesoderm genes.** (A) The expression of epiblast genes (T, Fgf5, and Dnmt3b) on day 2 of cardiomyocyte differentiation after Zhx1 knockout. (B) The expression of mesoderm genes (Flk1, Pdgfrα, Eomes, Mixl1, and Mesp1) on day 4 of cardiomyocyte differentiation after Zhx1 knockout. (C) The percentage of Flk1^+^/ Pdgfrα^+^ cells on day 4 of cardiomyocyte differentiation after Zhx1 knockout. (D) The expression of cardiac progenitor genes (Nkx2.5, Isl1, Mef2c, Tbx5, Hand1, and Hand2) on day 6 of cardiomyocyte differentiation after Zhx1 knockout. Data are presented as the mean ± SEM (n = 3). The statistical signiﬁcance is performed according to Student’s t-tests (unpaired two-tailed). *p < 0.05, **p < 0.01, and ***p < 0.001 versus 46C-Ctrl.


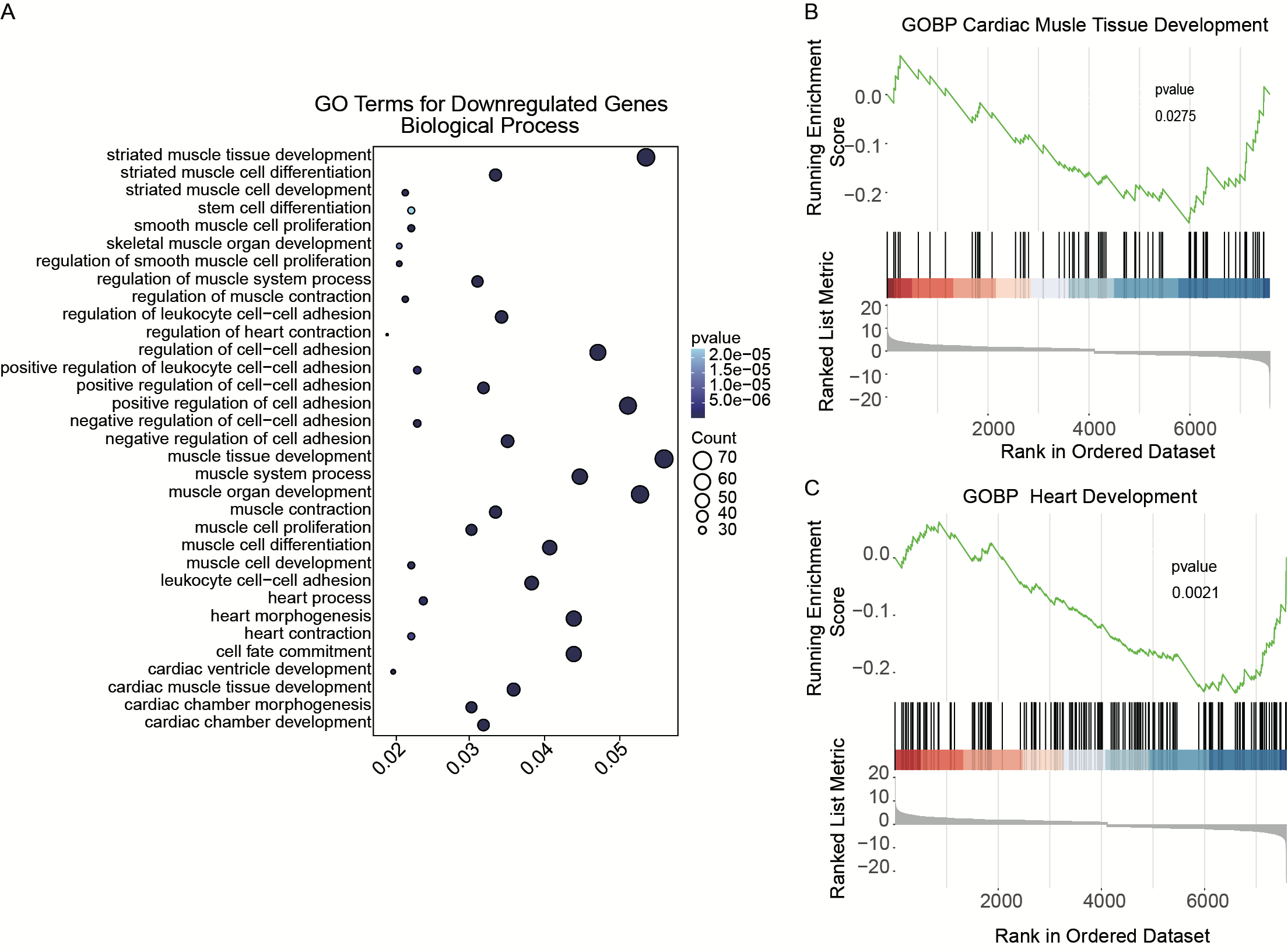


**Supplementary Fig. S5. GO Term analysis for genes after Zhx1 deletion.** (A) GO Term analysis for downregulated genes after Zhx1 knockout. (B and C) GSEA analysis for differentially expressed genes after Zhx1 knockout.


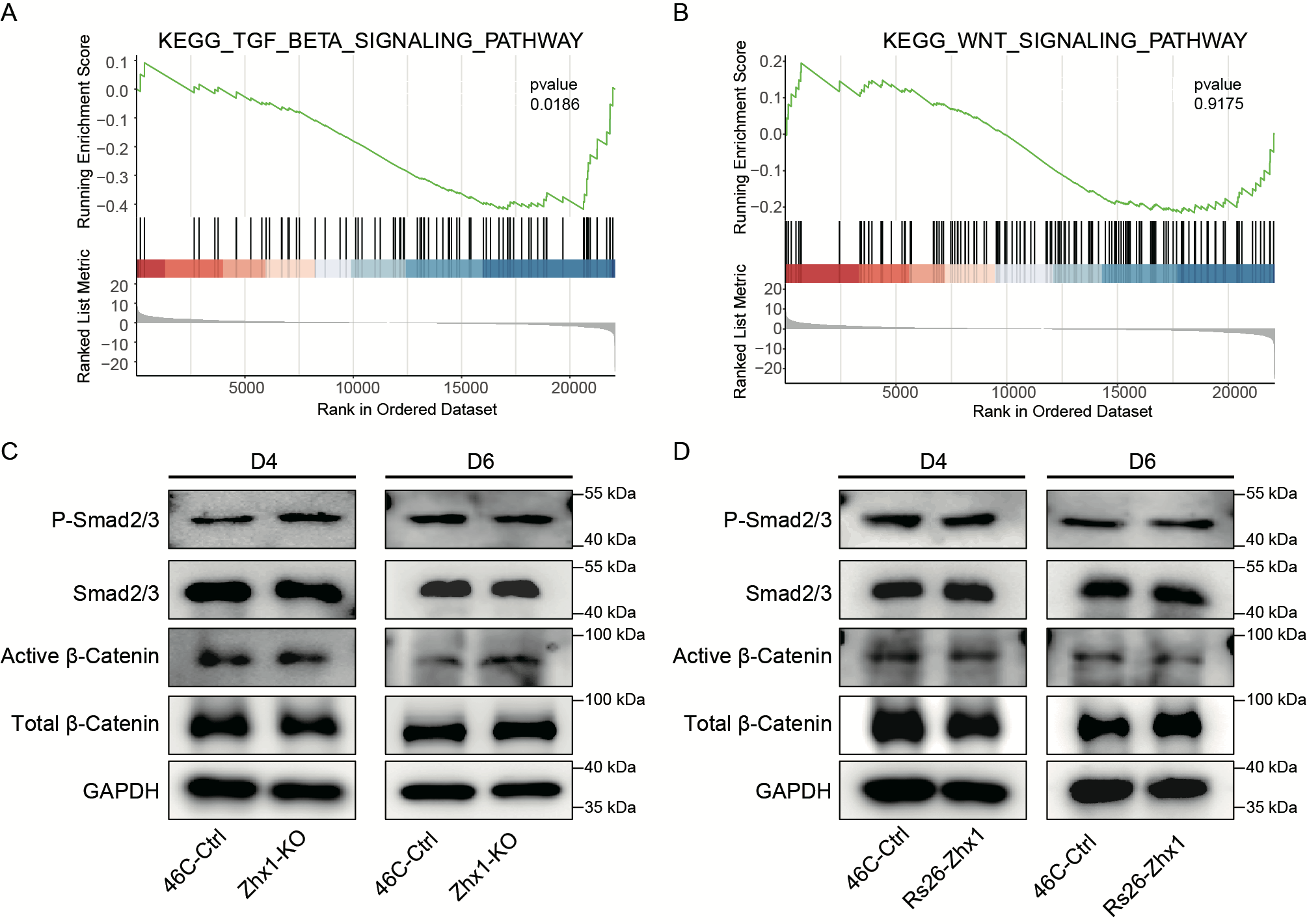


**Supplementary Fig. S6. Zhx1 does not affect the Wnt or Smad2/3 pathway in cardiomyocyte differentiation.** (A and B) The GSEA analysis of TGF-β (A) and Wnt (B) pathways after Zhx1 knockout. (C) The western blot results of protein expression of Wnt pathway effector β-Catenin (active-β-Catenin and total β-Catenin) and Smad2/3 (p-Smad2/3 and total Smad2/3) after Zhx1 knockout at day 4 or day 6 of cardiomyocyte differentiation. (D) The western blot results of protein expression of active-β-Catenin, total β-Catenin, p-Smad2/3, and total Smad2/3 after Zhx1 overexpression at day 4 or day 6 of cardiomyocyte differentiation.

**Supplemental Tables**

**Table S1. Primers used for vectors construction.**

| Fuw-Flag-Znf-Mut2-2 | CCCAATGTTGTGCTGAATTCCTCCCCAGGAGAAGAAAATTTCAAGCTGACTATG | F |
| --- | --- | --- |
|  | TGATTATCGATAAGCTTGATATCGTCAGTCATCCGATTTAGAAAGCTTCCGCTT | R |
| Fuw-Flag-Del111-120-1 | GCTGCAGGTCGACTCTAGAGATGGATTACAAGGATGACGACGATAAGGCAAGCAGACGAAAATCAACAAC | F |
|  | CAGATTATGGGTAAGAAAATTGCATTC | R |
| Fuw-Flag-Del111-120-2 | TCTTACCCATAATCTGAAATACCAC | F |
|  | TGATTATCGATAAGCTTGATATCGTCAGTCATCCGATTTAGAAAGCTTCCGCTT | R |
| Fuw-Flag-Del120-125-1 | GCTGCAGGTCGACTCTAGAGATGGATTACAAGGATGACGACGATAAGGCAAGCAGACGAAAATCAACAAC | F |
|  | CTGGGTGATGCTCAGAAAGTGCATCA | R |
| Fuw-Flag-Del120-125-2 | TGAGCATCACCCAGGAGAAGAAAAT | F |
|  | TGATTATCGATAAGCTTGATATCGTCAGTCATCCGATTTAGAAAGCTTCCGCTT | R |
| Zhx1-5’gRNA-1 | CACCGTCTCATTAGTAGCGTTTCCA | F |
|  | AAACTGGAAACGCTACTAATGAGAC | R |
| Zhx1-3’gRNA -1 | CACCGGAAGCGGAAGCTTTCTAAAT | F |
|  | AAACATTTAGAAAGCTTCCGCTTCC | R |
| SMASh-NotI-Zhx1-5’arm-F | CACAGGCGGCCGCACAATGACTCCTCTGCCACTATCATCATAGACT | F |
| SMASh-Zhx1-5’arm-R | CACGTCGTAGGGATAGTCATCCGATTTAGAAAGCTTCCGCTTC | R |
| SMASh-Zhx1-5’arm-F  SMASh-Zhx1-SalI-loxp-R | TCTAAATCGGATGACTATCCCTACGACGTGCCTGATTATGCAT |  |
|  |  | F |
|  | CACAGGTCGACATAACTTCGTATAATGTATGCTATACGAAGTTATTTAGTACAGCACCTCTCTATCGGGGATG | R |
| SMASh-Zhx1-loxp-3’arm | CACAGGAATTCATAACTTCGTATAGCATACATTATACGAAGTTATGTAAGTGTTTAAGCCTTCATGTTTATCACT | F |
|  | CACAGGAATTCTAGTTACTCAAAAACAGATGCCACCTAAGCACTG | R |
| Rs26-gRNA | CACCGCTGGAGTTGCAGATCACGA | F |
|  | AAACTCGTGATCTGCAACTCCAGC | R |
| SMASh-gRNA | CACCGTTTAGCAATACTAGGTGTTC | F |
|  | AAACGAACACCTAGTATTGCTAAAC | R |
| hZHX1-shRNA-1 | CCGG TCGGGAATATCTATCAGTAAA CTCGAG TTTACTGATAGATATTCCCGA TTTTTG | F |
|  | AATTCAAAAA TCGGGAATATCTATCAGTAAA CTCGAG TTTACTGATAGATATTCCCGA | R |
| hZHX1-shRNA-2 | CCGG CCAGTGATGAAACCACGGAAT CTCGAG ATTCCGTGGTTTCATCACTGG TTTTTG | F |
|  | AATTCAAAAA CCAGTGATGAAACCACGGAAT CTCGAG ATTCCGTGGTTTCATCACTGG | R |

**Table S2. Primers used for RT-qPCR and ChIP-qPCR assays.**

| Zhx1 | GCAAGCAGACGAAAATCAACAA | F |
| --- | --- | --- |
|  | TCTACAGGTGTAAGGATGGGAG | R |
| Hnrnpa1 | TGGAAGCAATTTTGGAGGTGG | F |
|  | GGTTCCGTGGTTTAGCAAAGT | R |
| Gapdh | GTGTTCCTACCCCCAATGTGT | F |
|  | ATTGTCATACCAGGAAATGAGCTT | R |
| Nanog | AGGGTCTGCTACTGAGATGCTCTG | F |
|  | CAACCACTGGTTTTTCTGCCACCG | R |
| Oct4 | GGATGCTGTGAGCCAAGG | F |
|  | GAACAAAATGATGAGTGACAGACAG | R |
| Sox2 | GCGGAGTGGAAACTTTTGTCC | F |
|  | CGGGAAGCGTGTACTTATCCTT | R |
| Esrrb | TGGCAGGCAAGGATGACAGA | F |
|  | TTTACATGAGGGCCGTGGGA | R |
| T | GCTCTAAGGAACCACCGGTCATC | F |
|  | ATGGACTGCAGCATGGACAG | R |
| Fgf5 | AAGTAGCGCGACGTTTTCTTC | F |
|  | CTGGAAACTGCTATGTTCCGAG | R |
| Dnmt3b | AGCGGGTATGAGGAGTGCAT | F |
|  | GGGAGCATCCTTCGTGTCTG | R |
| Eomes | CCCTATGGCTCAAATTCCAC | F |
|  | CCAGAACCACTTCCACGAAA | R |
| Mixl1 | TCCAGGATCCAGGTGTGGTT | F |
|  | GCAGGGCAATGGAGGAAAAC | R |
| Mesp1 | GTTCCTGTACGCAGAAACAGCATC | F |
|  | CAAGGAGGGTTGGAATGGTACAGT | R |
| Flk1 | TTTGGCAAATACAACCCTTCAGA | F |
|  | GCAGAAGATACTGTCACCACC | R |
| Pdgfrα | CACACCGGATGGTACACTTG | F |
|  | GGCAGAGTCATCCTCTTCCA | R |
| Nkx2.5 | GACAGGTACCGCTGTTGCTT | F |
|  | AGCCTACGGTGACCCTGAC | R |
| Tbx5 | TGACTGGCCTTAATCCCAAA | F |
|  | ACAAGTTGTCGCATCCAGTG | R |
| Mef2c | AGCAAGAATACGATGCCATC | F |
|  | GAAGGGGTGGTGGTACGGTC | R |
| Isl1 | TGTCAGGAGACTTGCCACTTT | F |
|  | GCCAAACGTTTATTAGTGAAATAGT | R |
| Hand1 | GCCTACTTGATGGACGTGCT | F |
|  | GTGCGCCCTTTAATCCTCTT | R |
| Hand2 | ACCAGCTACATCGCCTACCT | F |
|  | GCTTTTCAAGATCTCATTCAGC | R |
| Myl2 | AAAGAGGCTCCAGGTCCAAT | F |
|  | CCTCTCTGCTTGCGTGGTTA | R |
| Myl7 | CCCATCAACTTCACCGTCTTCCT | F |
|  | AGAGAACTTGTCTGCCTGGGTCA | R |
| α-MHC | CAAGACTGTCCGGAATGACA | F |
|  | GGCTTCTTGTTGGACAGGAT | R |
| β-MHC | ACTGTCAACACTAAGAGGGTCA | F |
|  | TTGGATGATTTGATCTTCCAGGG | R |
| Slc8a1 | AGGTCCATGCTAGAGATCATCC | F |
|  | CATCATCGTCATCTTCCCCA | R |
| cTnT | CAGAGGAGGCCAACGTAGAAG | F |
|  | CTCCATCGGGGATCTTGGGT | R |
| hGapdh | GGAGCGAGATCCCTCCAAAAT | F |
|  | GGCTGTTGTCATACTTCTCATGG | R |
| hcTnT | AAGAAGAAGATTCTGGCTGAGAG | F |
|  | ACTTTCTGGTTATCGTTGATCCT | R |
| hMyh6 | TCAGCTGGAGGCCAAAAGTAAAGGA | F |
|  | TTCTTGAGCTCTGAGCACTCGTCT | R |
| hMyl7 | ACATCATCACCCATGGAGACGAGA | F |
|  | GCAACAGAGTTTATTGAGGTGCCC | R |
| hSlc8a1 | GACCTCGGTCCTAGCACCAT | F |
|  | ACACCAGGAGATATGACAGACAA | R |
| Cola1 | GCTCCTCTTAGGGGCCACT | F |
|  | CCACGTCTCACCATTGGGG | R |
| Col3a1 | GCTCCTCTTAGGGGCCACT | F |
|  | CCACGTCTCACCATTGGGG | R |
| Ckap4 | TCCCGTCAGAGGGATGAGC | F |
|  | GCTGGGAGTTTCTCAGGAGG | R |
| Pecam1 | ACGCTGGTGCTCTATGCAAG | F |
|  | TCAGTTGCTGCCCATTCATCA | R |
| Vecad | GTCGATGCTAACACAGGGAATG | F |
|  | AATACCTGGTGCGAAAACACA | R |
| Cnn1 | AAACAAGAGCGGAGATTTGAGC | F |
|  | TGTCGCAGTGTTCCATGCC | R |
| Des | GTGGATGCAGCCACTCTAGC | F |
|  | TTAGCCGCGATGGTCTCATAC | R |
| ChIP_*Mef2c*_Pro | ATGCGTGGTGCCAGTAAGTT | F |
|  | AGAAGCCACCGCTTTCCTTT | R |
| ChIP_*Src*_Pro | TTGGGGATACCCGACCTCTT | F |
|  | TCCCTGCAATGGATGAGAGA | R |
| ChIP_*Epas1*_Pro | CTGCCATGGGACAACTCTGT | F |
|  | CTCAACCCCCTACAAGGCTG | R |

**Table S3. Antibodies used in this study.**

| **Antibodies** | **Manufacture** | **Catalog** |
| --- | --- | --- |
| Anti-ZHX1 | Novus | NB600-244 |
| Anti-hnRNPA1 | Novus | NB100-672 |
| Anti-Flag | Cell Signaling Technology | 14793s |
| Anti-HA | Cell Signaling Technology | 5017S |
| Anti-Smad2/3 | Cell Signaling Technology | 5678s |
| Anti-Rabbit HRP | Cell Signaling Technology | 7074S |
| Anti-Mouse HRP | Cell Signaling Technology | 7076S |
| Anti-Active β-Catenin | Millipore | 05665 |
| Ani-cTNT | Abcam | ab8295 |
| Anti-Total β-Catenin | Abcam | ab16051 |
| Anti-GAPDH | Bioworld | AP0063 |
| Anti-p-Smad2/3 | Bioworld | BS1838 |
| Donkey anti-Rabbit IgG 594 | Invitrogen | A21207 |
| Donkey anti-Rabbit IgG 488 | Invitrogen | A21206 |
| Donkey anti-Mouse IgG 594 | Invitrogen | A21203 |
| Donkey anti-Mouse IgG 488 | Invitrogen | A21202 |
| Anti-α-Actinin | Sigma | A7811 |
| Hoechst33342 | Sigma | 14533 |

**Table S4. The protein interacted with ZHX1 identified by mass spectrometry assay**.

| Gene | Proteinscore | coverage | Unique |
| --- | --- | --- | --- |
| Hsd17b4 | 536.39 | 18.78 | 11 |
| Krt16 | 246.14 | 8.32 | 2 |
| Ywhaq | 161.09 | 15.1 | 2 |
| Hnrnpa1 | 139.69 | 9.69 | 2 |
| Ybx3 | 127.32 | 11.36 | 2 |
| Mff | 121.45 | 7.22 | 2 |
| Dync1i2 | 110.66 | 7.52 | 3 |
| Rplp2 | 102.1 | 21.74 | 2 |
| Lsp1 | 87.57 | 6.67 | 2 |
| D1Pas1 | 80.29 | 3.48 | 2 |
| H3-5 | 75.33 | 10.29 | 2 |
| Rpl3 | 74.24 | 5.21 | 2 |
| Cul5 | 73.11 | 2.95 | 2 |
| Rps13 | 70.09 | 11.26 | 2 |
| Pabpc1 | 69.3 | 2.67 | 2 |
| Rnmt | 63.81 | 4.73 | 2 |
