# Supplementary material for: Cooperative regulation of Zhx1 and hnRNPA1 drives the cardiac progenitor-specific transcriptional activation during cardiomyocyte differentiation
Source: Cell Death Discov. 2023 Jul 14;9:244. doi: 10.1038/s41420-023-01548-1 (PMC10349095; doi:10.1038/s41420-023-01548-1)

Supplemental Data

Original western blots

Figure 1F. Anti-ZHX1

Figure 1F. Anti-GAPDH

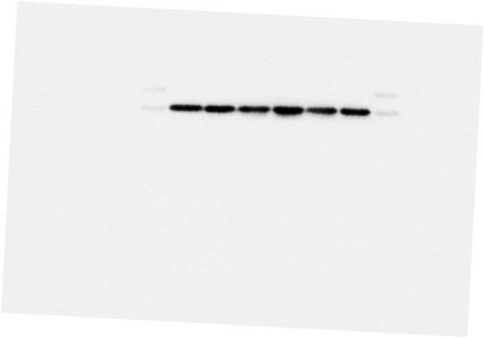

Figure 1H. Anti-cTNT

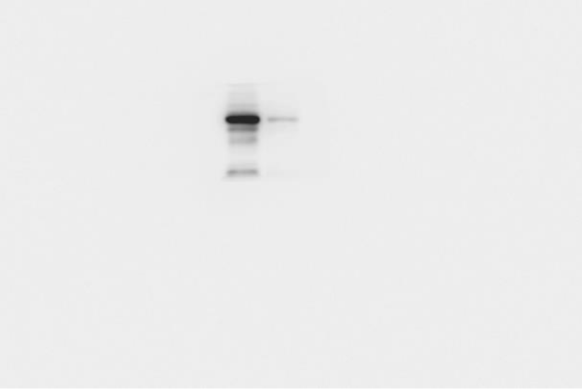

Figure 1H. Anti-α-ACTININ

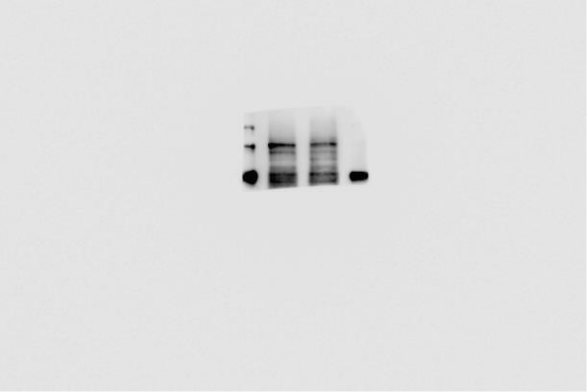

Figure1H. Anti-GAPDH

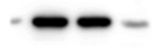

Figure 2B. Anti-ZHX1

Figure 2B. Anti-GAPDH

Figure 2E. Anti-ZHX1 D2

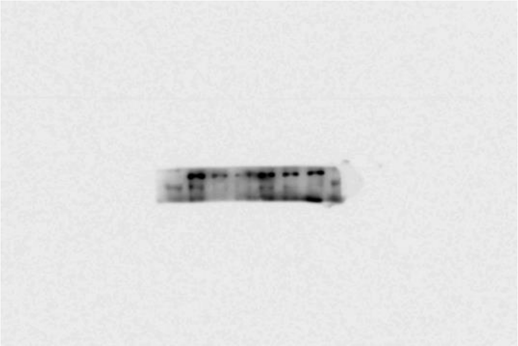

Figure 2E. Anti-GAPDH D2

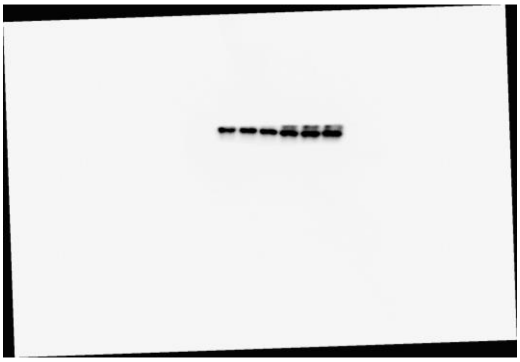

Figure 2E. Anti-ZHX1 D4

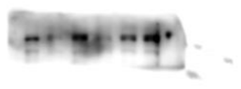

Figure 2E. Anti-GAPDH D4

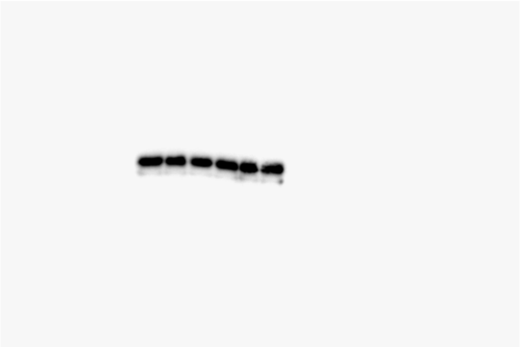

Figure 2E. Anti-ZHX1 D6

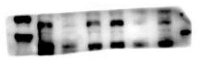

Figure 2E. Anti-GAPDH D6

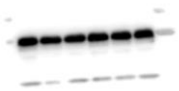

Figure 2E. Anti-ZHX1 D8

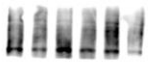

Figure 2E. Anti-GAPDH D8

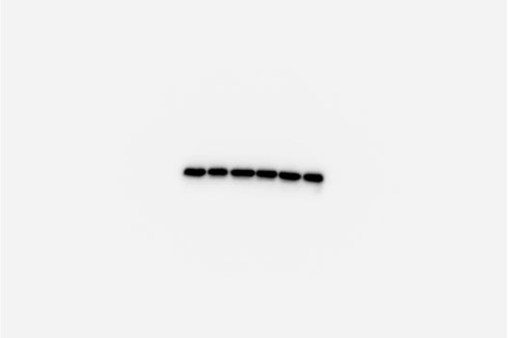

Figure 2G. Anti-cTNT

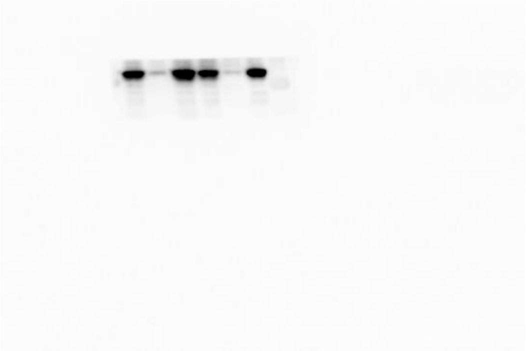

Figure 2G. Anti-α-ACTININ

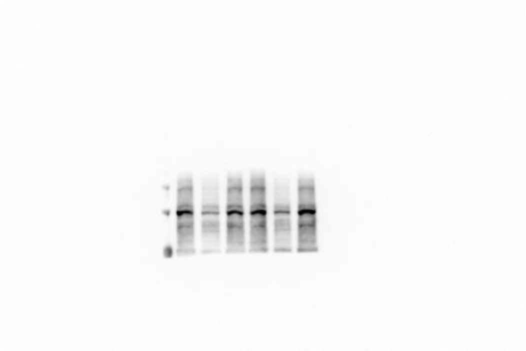

Figure 2G. Anti-GAPDH

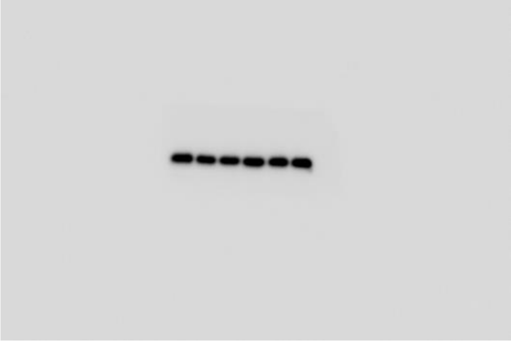

Figure 3C. Anti-ZHX1

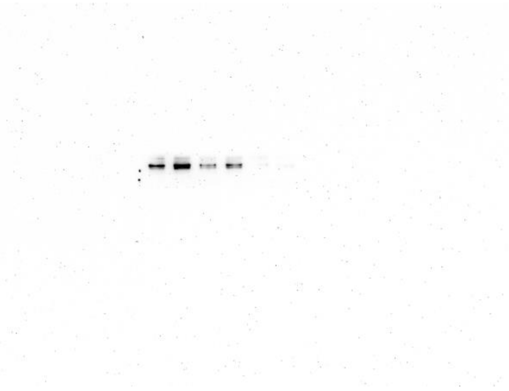

Figure 3C. Anti-GAPDH

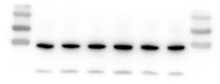

Figure 3J. Anti-cTNT

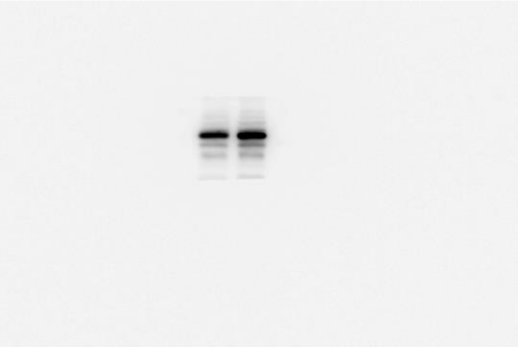

Figure 3J. Anti-α-ACTININ

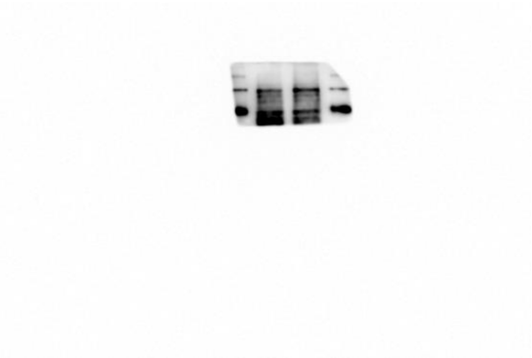

Figure 3J. Anti-GAPDH

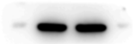

Figure 4B. Anti-FLAG

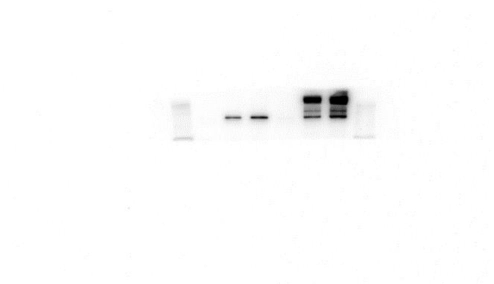

Figure 4B. Anti-HA

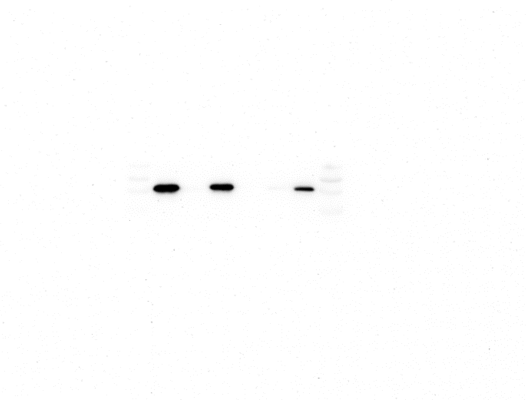

Figure 4B. Anti-FLAG

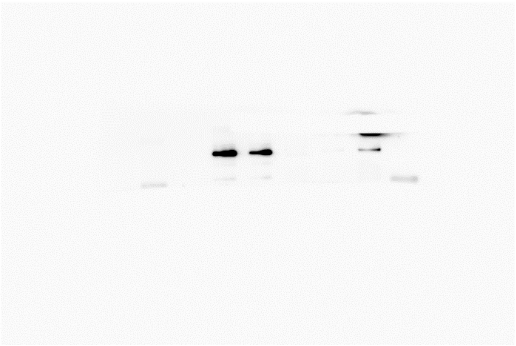

Figure 4B. Anti-HA

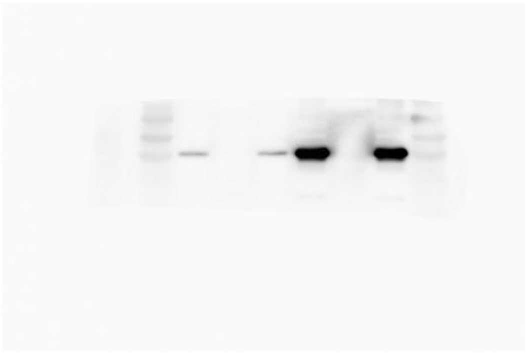

Figure 5B. Input: Anti-Flag

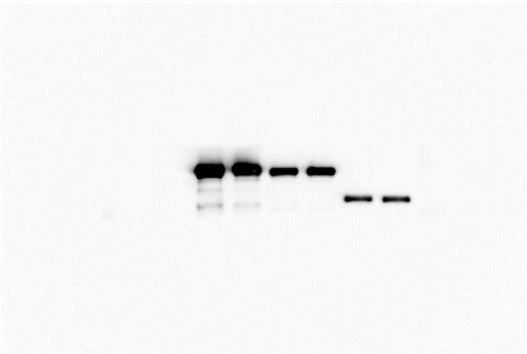

Figure 5B. Input: Anti-HA

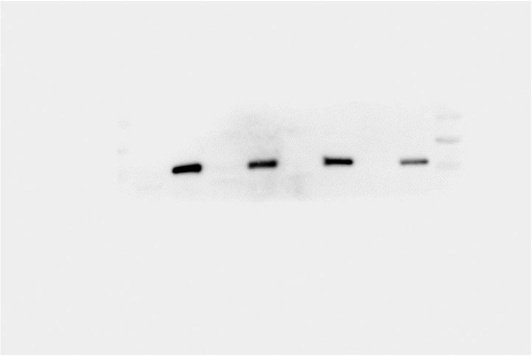

Figure 5B. IP: Anti-Flag

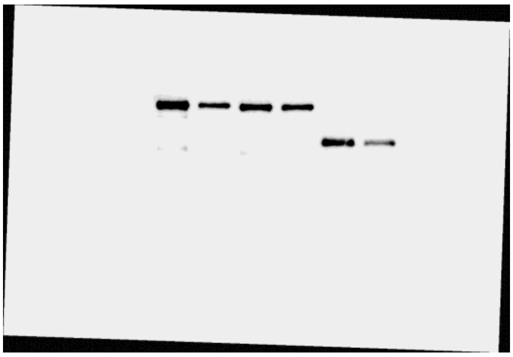

Figure 5B. IP: Anti-HA

Figure 5D. Input: Anti-HA

Figure 5D. Input: Anti-Flag

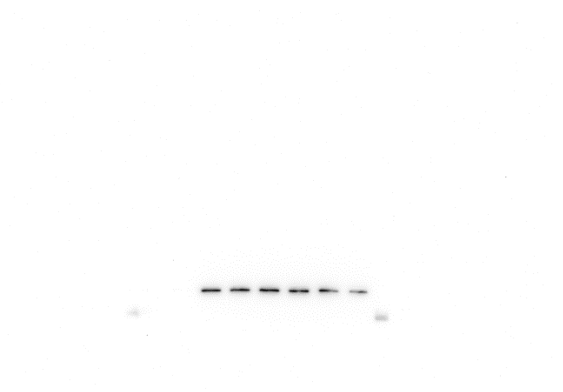

Figure 5D. IP: Anti-HA

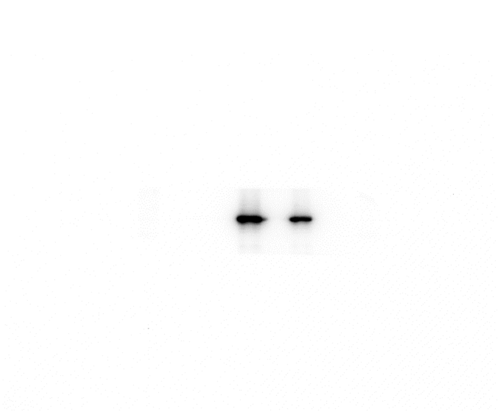

Figure 5D. IP: Anti-Flag

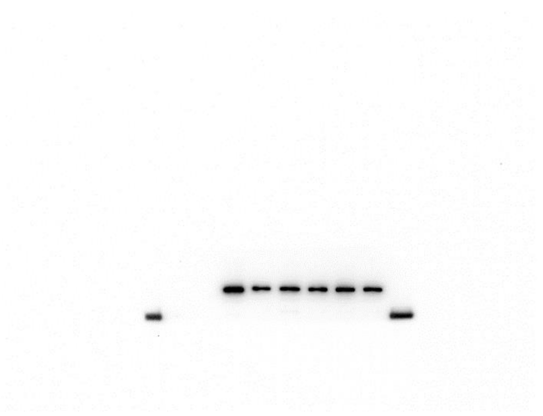

Figure 5E. Input: Anti-Flag

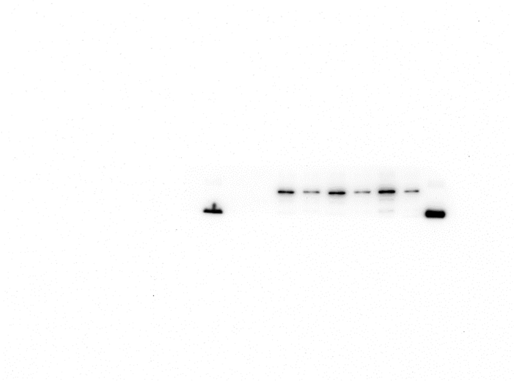

Figure 5E. Input: Anti-HA

Figure 5E. IP: Anti-Flag

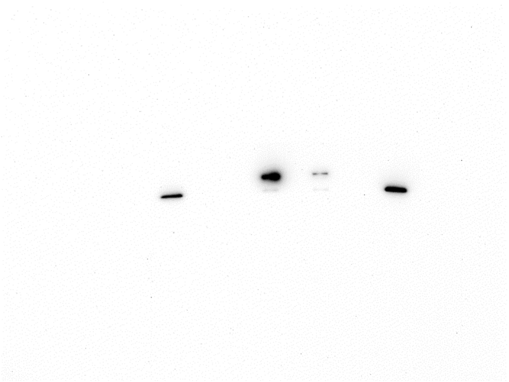

Figure 5E. IP: Anti-HA

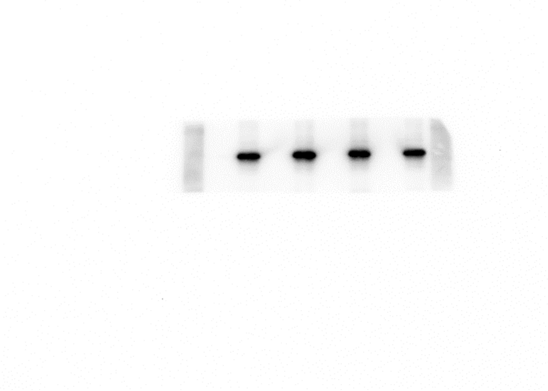

Figure 5G. Input: Anti-Flag

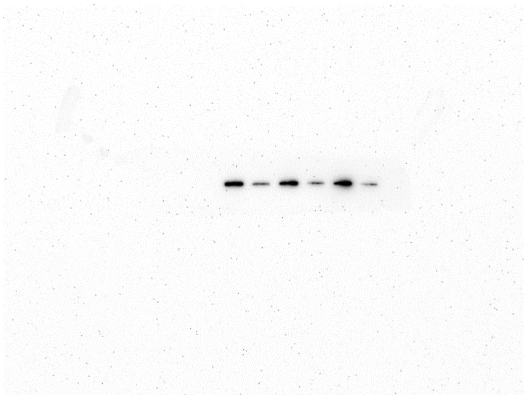

Figure 5G. Input: Anti-HA

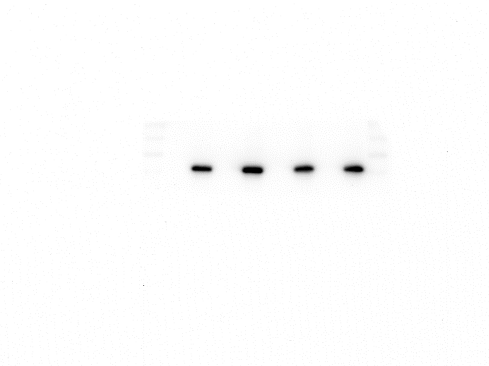

Figure 5G. IP: Anti-Flag

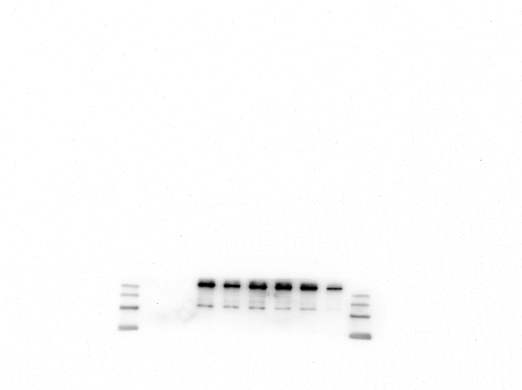

Figure 5G. IP: Anti-HA

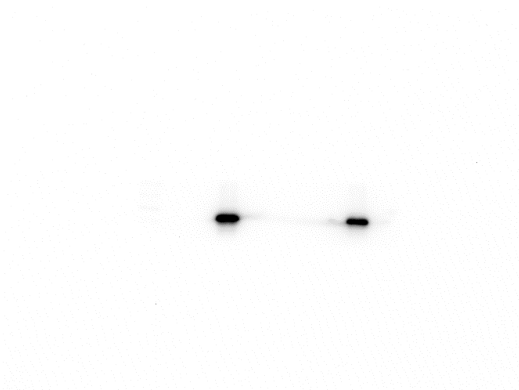

Figure 5H. Input: Anti-Flag

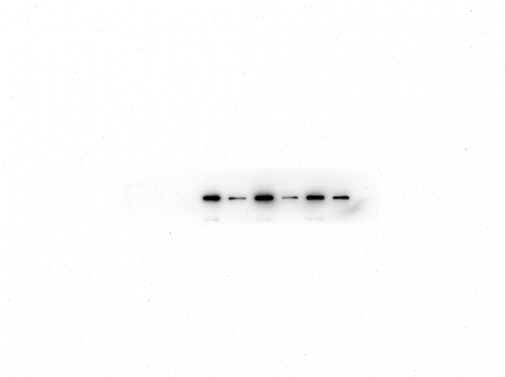

Figure 5H. Input: Anti-HA

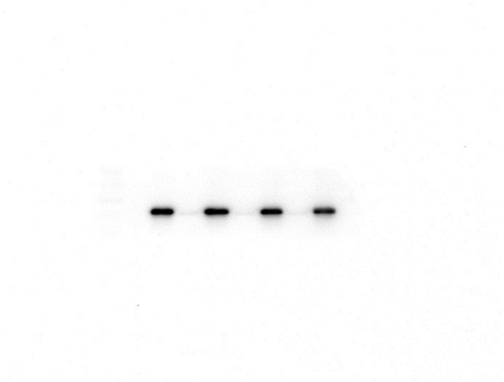

Figure 5H. IP: Anti-Flag

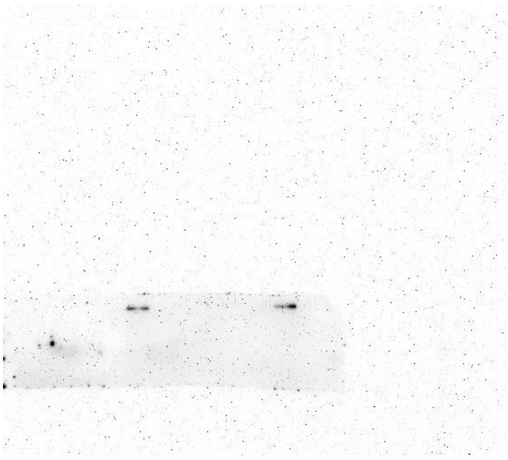

Figure 5H. IP: Anti-HA

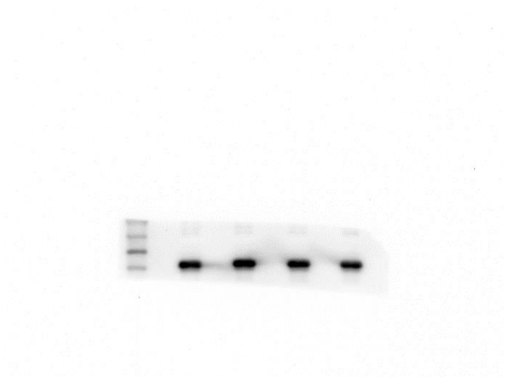

Figure 6B. Anti-ZHX1

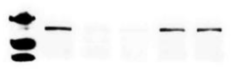

Figure 6B. Anti-GAPDH

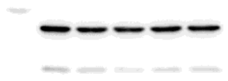

Figure 6E. Anti-cTNT

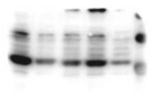

Figure 6E. Anti-α-ACTININ

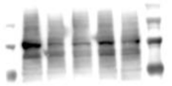

Figure 6E. Anti-GAPDH

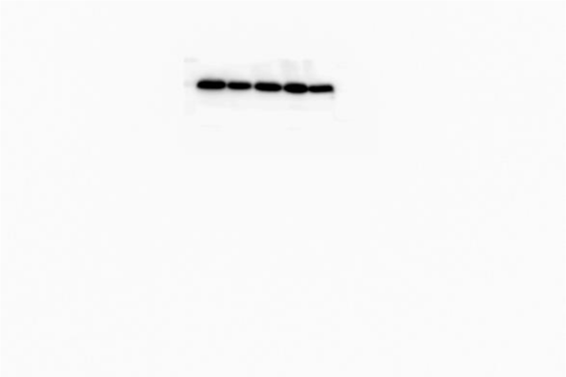

Figure S3A. Anti-ZHX1

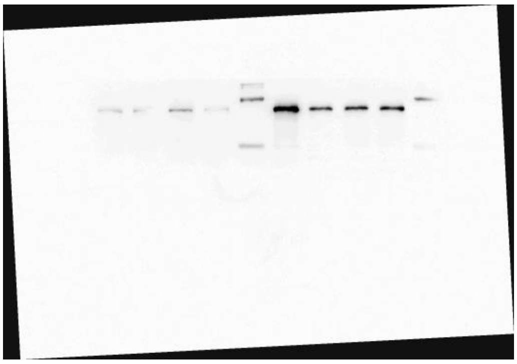

Figure S3A. Anti-GAPDH

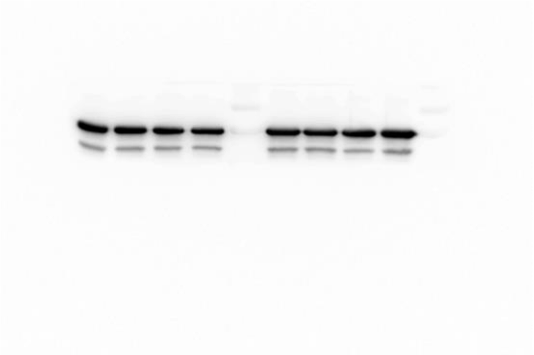

Figure S3C. Anti-CTNT

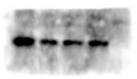

Figure S3C. Anti-α-ACTININ

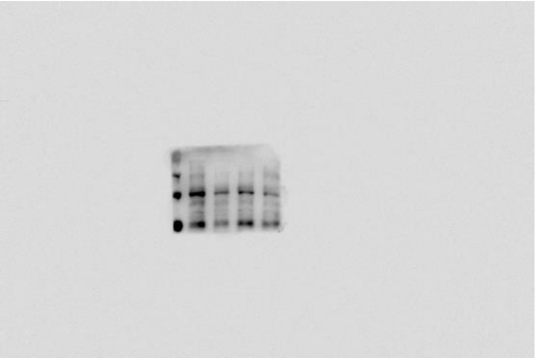

Figure S3C. Anti-GAPDH

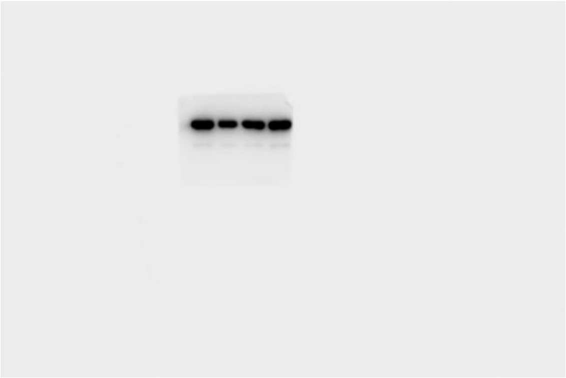

Figure S6C. Anti-P-Smad2/3 D4

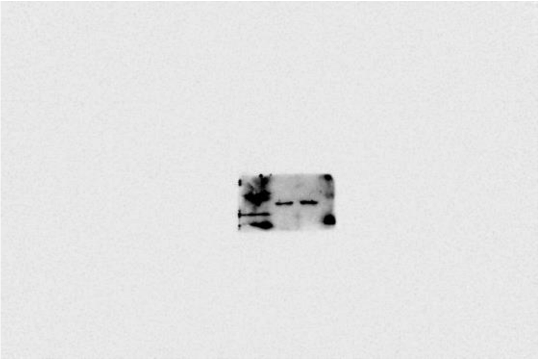

Figure S6C. Anti- Smad2/3 D4

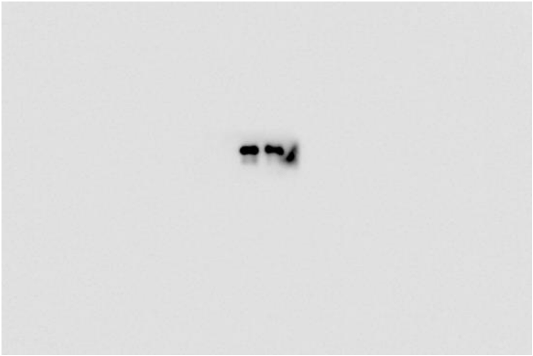

Figure S6C. Anti-Active β-Catenin D4

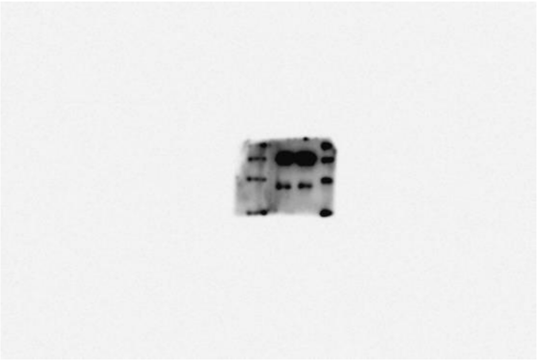

Figure S6C. Anti-Total β-Catenin D4

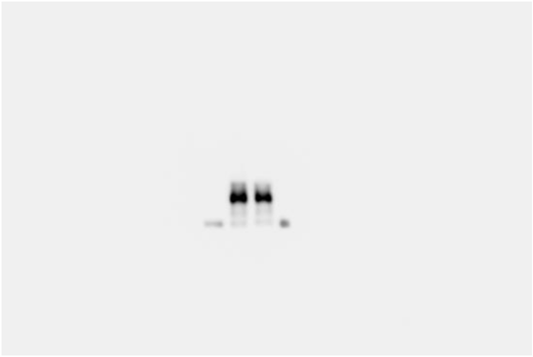

Figure S6C. Anti-GAPDH D4

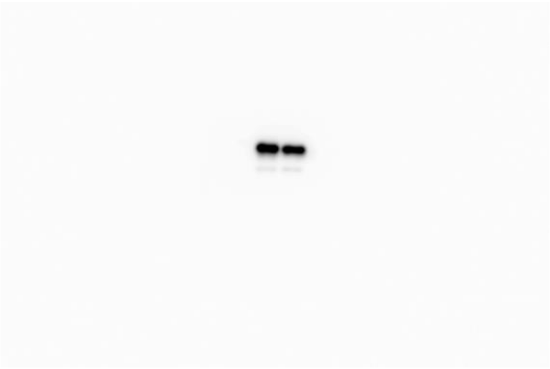

Figure S6C. Anti-P-Smad2/3 D6

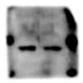

Figure S6C. Anti- Smad2/3 D6

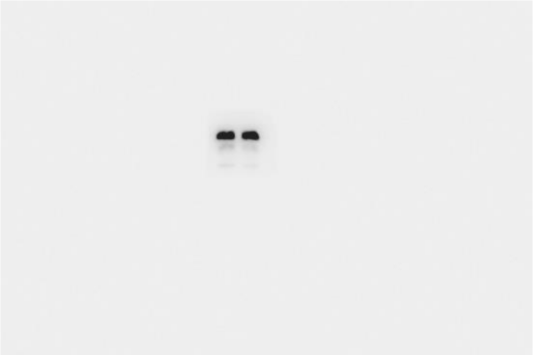

Figure S6C. Anti-Active β-Catenin D6

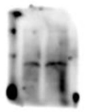

Figure S6C. Anti-Total β-Catenin D6

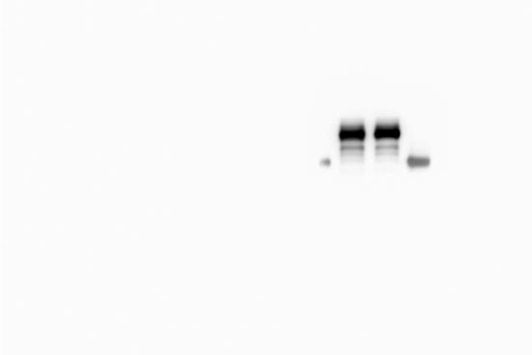

Figure S6C. Anti-GAPDH D6

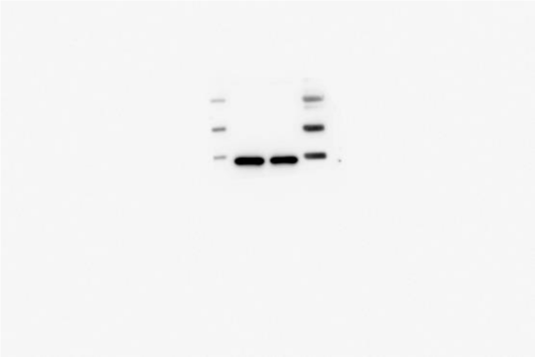

Figure S6D. Anti-P-Smad2/3 D4

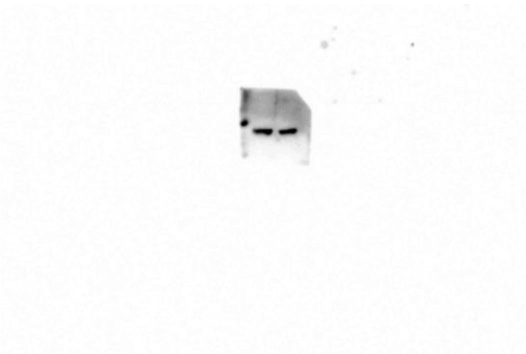

Figure S6D. Anti- Smad2/3 D4

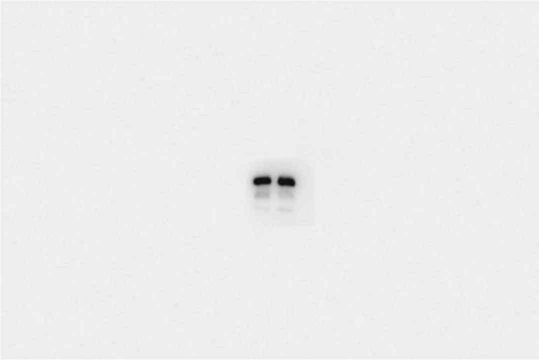

Figure S6D. Anti-Active β-Catenin D4

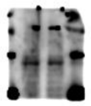

Figure S6D. Anti-Total β-Catenin D4

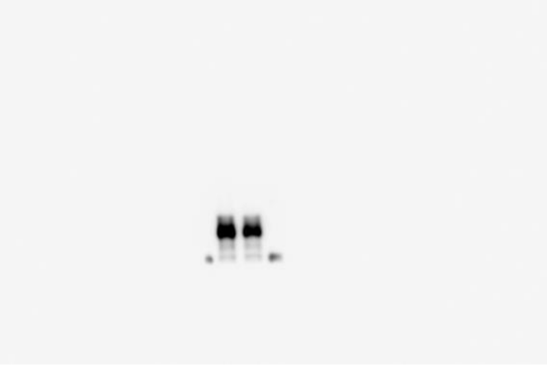

Figure S6D. Anti-GAPDH D4

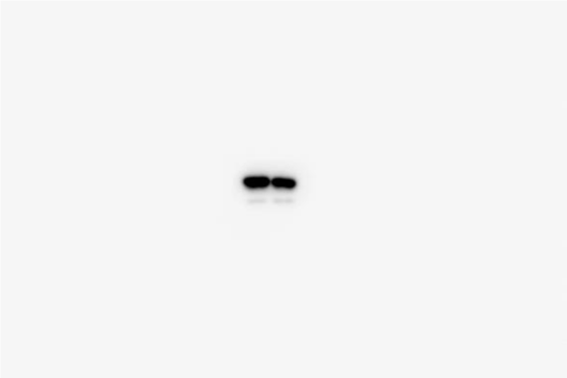

Figure S6D. Anti-P-Smad2/3 D6

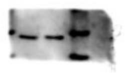

Figure S6D. Anti- Smad2/3 D6

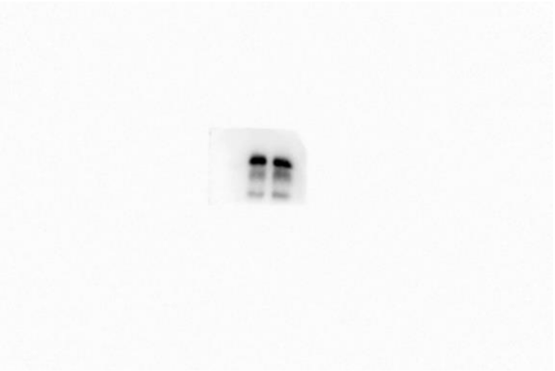

Figure S6D. Anti-Active β-Catenin D6

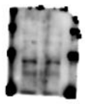

Figure S6D. Anti-Total β-Catenin D6

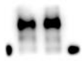

Figure S6D. Anti-GAPDH D6

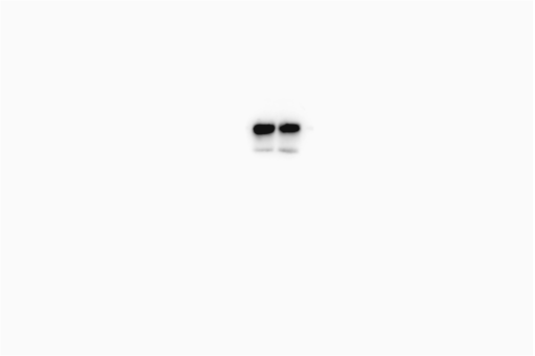

Supplement: Supplementary file 2 — Original Data File [file 41420_2023_1548_MOESM2_ESM.pdf]
