# Supplementary material for: Cooperative regulation of Zhx1 and hnRNPA1 drives the cardiac progenitor-specific transcriptional activation during cardiomyocyte differentiation
Source: Cell Death Discov. 2023 Jul 14;9:244. doi: 10.1038/s41420-023-01548-1 (PMC10349095; doi:10.1038/s41420-023-01548-1)
Supplement: Supplementary file 3 — Co-author agreement [file 41420_2023_1548_MOESM3_ESM.pdf]

Co-authors' email responses

(1) Agreement from Mr. Yang Chen

Dear Prof. Guo,

Thanks for your Email. I agree to these changes of adding two authors to the author li sts of manuscript (DDISCOVERY-23-0180R1).

------------------ 原始邮件 ------------------
发件人: "郭旭东"<19504@tongji.edu.cn>;
发件时间: 2023-07-05 11:49
收件人: "825645918"<825645918@qq.com>;
主题: Asking to obtain the agreement about the author addition

Dear Mr. Yang Chen,
This E-mail is asking to obtain your agreement about the additions of the following authors (Mr Jianguo Li and Mr Ke Feng) to the author lists of manuscript (DDISCOVERY-23-0180R1). And you might reply to this email and confirm that you are agree to these changes.

Best wishes,
Xudong Guo

Asking to obtain the agreement about the author addition

Dear Prof. Guo,

Thanks for your E-mail. I agree to these changes of adding two authors to the author lists of manuscript (DDISCOVERY-23-0180R1).

------------------ 原始邮件 ------------------
发件人: "郭旭东"<19504@tongji.edu.cn>;
发件时间: 2023-07-05 11:49
收件人: "825645918"<825645918@qq.com>;
主题: Asking to obtain the agreement about the author addition

Dear Mr. Yang Chen,
This E-mail is asking to obtain your agreement about the additions of the following authors (Mr Jianguo Li and Mr Ke Feng) to the author lists of manuscript (DDISCOVERY-23-0180R1). And you might reply to this email and confirm that you are agree to these changes.

Best wishes,
Xudong Guo

(2) Agreement from Dr. Yukang Wu

Dear Prof. Guo,

Thanks for your E-mail. I agree to these changes of adding two authors to the author lists of manuscript (DDISCOVERY-23-0180R1).

Yukang Wu
20230705

> 2023 年 7 月 5 日 11:42，郭旭东 <19504@tongji.edu.cn> 写道：
>
> Dear Dr. Yukang Wu,
> This E-mail is asking to obtain your agreement about the additions of the following authors (Mr Jianguo Li and Mr Ke Feng) to the author lists of manuscript (DDISCOVERY-23-0180R1). And you might reply to this email and confirm that you are agree to these changes.
>
> Best wishes,
> Xudong Guo

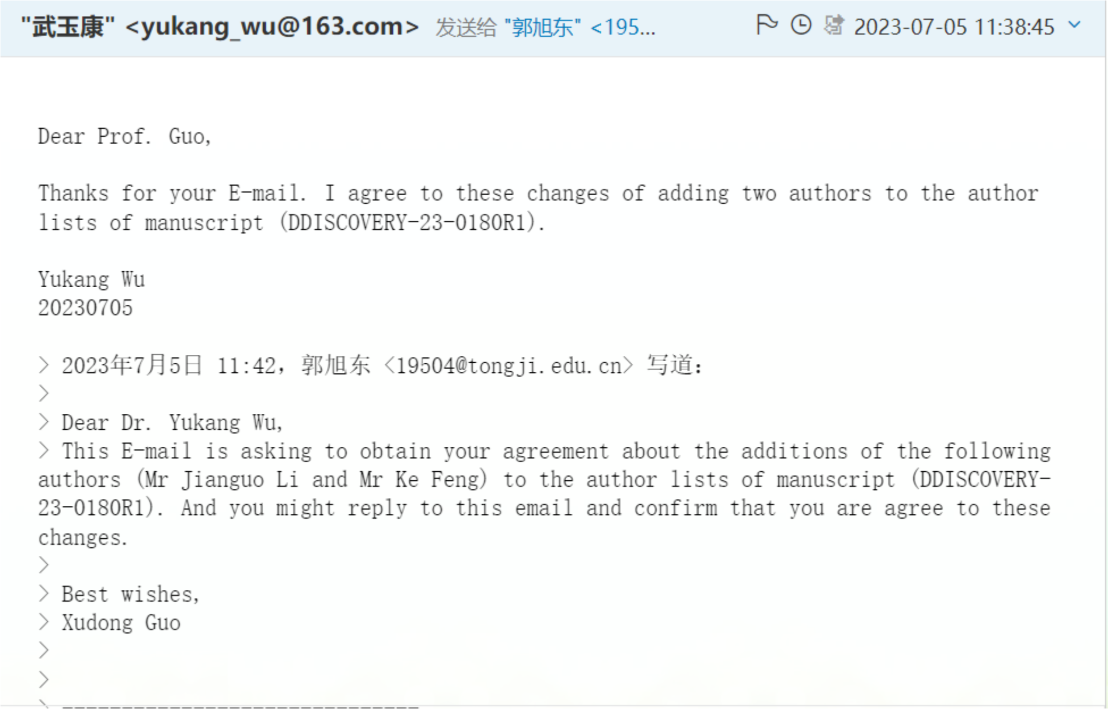

"武玉康" <yukang_wu@163.com> 发送给 "郭旭东" <195... 2023-07-05 11:38:45

Dear Prof. Guo,

Thanks for your E-mail. I agree to these changes of adding two authors to the author lists of manuscript (DDISCOVERY-23-0180R1).

Yukang Wu
20230705

> 2023年7月5日 11:42，郭旭东 <19504@tongji.edu.cn> 写道：
>
> Dear Dr. Yukang Wu,
> This E-mail is asking to obtain your agreement about the additions of the following authors (Mr Jianguo Li and Mr Ke Feng) to the author lists of manuscript (DDISCOVERY-23-0180R1). And you might reply to this email and confirm that you are agree to these changes.
>
> Best wishes,
> Xudong Guo
>
>
> -------------------------------

(3) Agreement from Mr. Jianguo Li

Dear Prof. Guo,
Thanks for your E-mail. I agree to these changes of adding two authors to the author lists of manuscript (DDISCOVERY-23-0180R1).

Best wishes,
Jianguo Li

> -----原始邮件-----
> 发件人: "郭旭东" <19504@tongji.edu.cn>
> 发送时间: 2023-07-05 11:42:49 (星期三)
> 收件人: lijianguo@tongji.edu.cn
> 抄送:
> 主题: Asking to obtain the agreement about the author addition
>
> Dear Mr. Jianguo Li,
> This E-mail is asking to obtain your agreement about the additions of the following authors (Mr Jianguo Li and Mr Ke Feng) to the author lists of manuscript (DDISCOVERY-23-0180R1). And you might reply to this email and confirm that you are agree to these changes.
>
> Best wishes,
> Xudong Guo

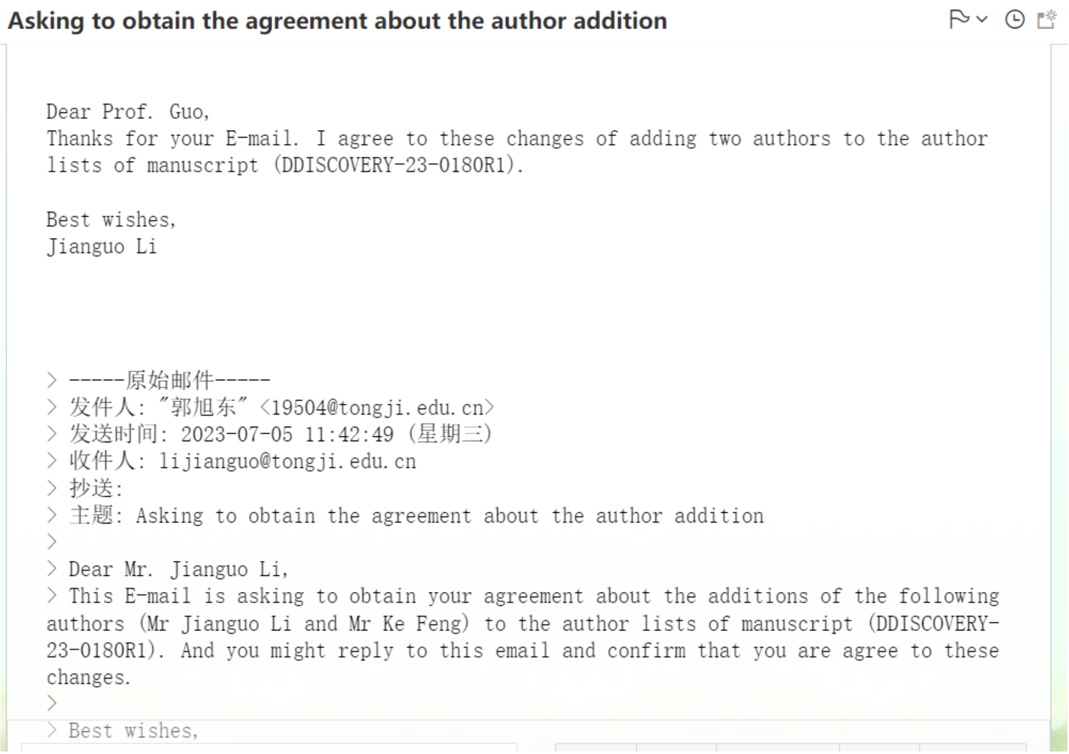

Asking to obtain the agreement about the author addition

Dear Prof. Guo,
Thanks for your E-mail. I agree to these changes of adding two authors to the author lists of manuscript (DDISCOVERY-23-0180R1).

Best wishes,
Jianguo Li

> -----原始邮件-----
> 发件人: "郭旭东" <19504@tongji.edu.cn>
> 发送时间: 2023-07-05 11:42:49 (星期三)
> 收件人: lijianguo@tongji.edu.cn
> 抄送:
> 主题: Asking to obtain the agreement about the author addition
>
> Dear Mr. Jianguo Li,
> This E-mail is asking to obtain your agreement about the additions of the following authors (Mr Jianguo Li and Mr Ke Feng) to the author lists of manuscript (DDISCOVERY-23-0180R1). And you might reply to this email and confirm that you are agree to these changes.
>
> Best wishes,

(4) Agreement from Mr. Kai Chen

Dear Prof. Guo,

Thanks for your E-mail. I agree to these changes of adding two authors to the author lists of manuscript (DDISCOVERY-23-0180R1).

Kai Chen

At 2023-07-05 10:44:14, "郭旭东" <19504@tongji.edu.cn> wrote:
>Dear Mr. Kai Chen,
>This E-mail is asking to obtain your agreement about the additions of the following authors (Mr Jianguo Li and Mr Ke Feng) to the author lists of manuscript (DDISCOVERY-23-0180R1). And you might reply to this email and confirm that you are agree to these changes.
>
>Best wishes,
>Xudong Guo

回复：Asking to obtain the agreement about the author addition

Dear Prof. Guo,

Thanks for your E-mail. I agree to these changes of adding two authors to the author lists of manuscript (DDISCOVERY-23-0180R1).

Kai Chen

At 2023-07-05 10:44:14, ″郭旭东″ <19504@tongji.edu.cn> wrote:
>Dear Mr. Kai Chen,
>This E-mail is asking to obtain your agreement about the additions of the following aut
>
>Best wishes,
>Xudong Guo

(5) Agreement from Mr. Wuchan Wang

Dear Prof. Guo,

Thanks for your E-mail. I agree to these changes of adding two authors to the author lists of manuscript (DDISCOVERY-23-0180R1).

Wuchan Wang

> -----原始邮件-----
> 发件人: "郭旭东" <19504@tongji.edu.cn>
> 发送时间: 2023-07-05 11:44:49 (星期三)
> 收件人: wangwuchan2019@tongji.edu.cn
> 抄送:
> 主题: Asking to obtain the agreement about the author addition
>
> Dear Mr. Wuchan Wang,
> This E-mail is asking to obtain your agreement about the additions of the following authors (Mr Jianguo Li and Mr Ke Feng) to the author lists of manuscript (DDISCOVERY-23-0180R1). And you might reply to this email and confirm that you are agree to these changes.
>
> Best wishes,
> Xudong Guo

Asking to obtain the agreement about the author addition

Dear Prof. Guo,
Thanks for your E-mail. I agree to these changes of adding two authors to the author lists of manuscript (DDISCOVERY-23-0180R1).
Wuchan Wang

> -----原始邮件-----
> 发件人: "郭旭东" <19504@tongji.edu.cn>
> 发送时间: 2023-07-05 11:44:49 (星期三)
> 收件人: wangwuchan2019@tongji.edu.cn
> 抄送:
> 主题: Asking to obtain the agreement about the author addition
>
> Dear Mr. Wuchan Wang,
> This E-mail is asking to obtain your agreement about the additions of the following authors (Mr Jianguo Li and Mr Ke Feng) to the author lists of manuscript (DDISCOVERY-23-0180R1). And you might reply to this email and confirm that you are agree to these changes.
>
> Best wishes,
> Xudong Guo
>
>

(6) Agreement from Miss. Zihui Ye

Dear Prof. Guo,
Thanks for your E-mail. I agree to these changes of adding two authors to the author lists of manuscript (DDISCOVERY-23-0180R1).

Zihui Ye

2023 年 7 月 5 日 11:45，郭旭东 <19504@tongji.edu.cn> 写道：

Dear Zihui Ye,
This E-mail is asking to obtain your agreement about the additions of the following authors (Mr Jianguo Li and Mr Ke Feng) to the author lists of manuscript (DDISCOVERY-23-0180R1). And you might reply to this email and confirm that you are agree to these changes.

Best wishes,
Xudong Guo

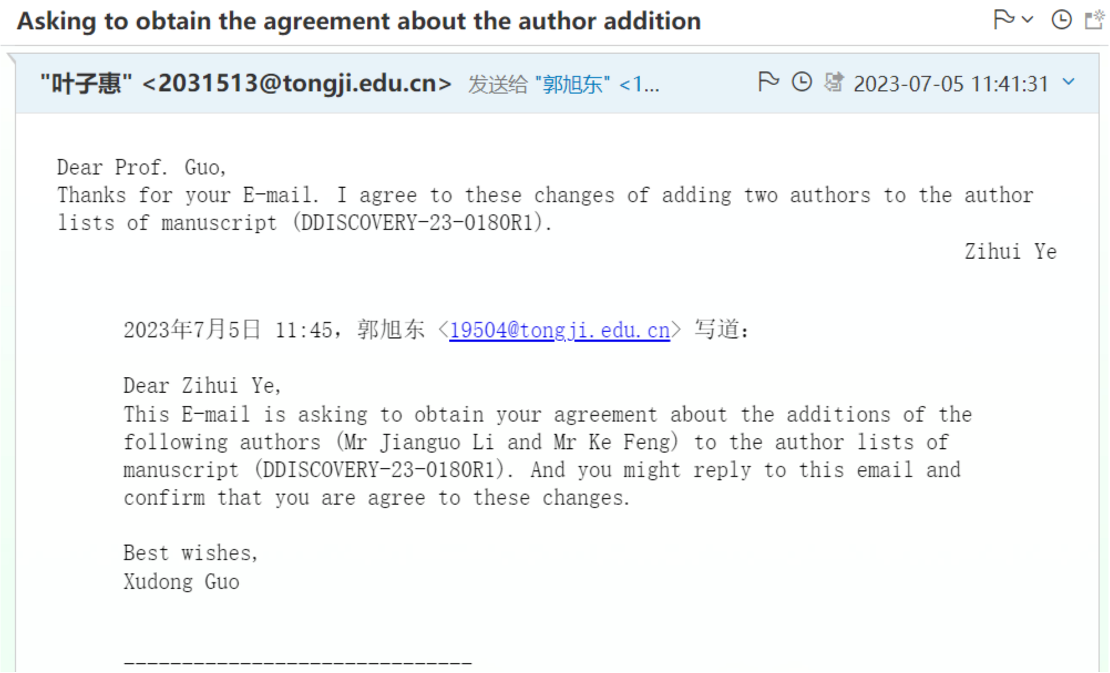
Asking to obtain the agreement about the author addition

"叶子惠" <2031513@tongji.edu.cn> 发送给 "郭旭东" <1... 2023-07-05 11:41:31

Dear Prof. Guo,
Thanks for your E-mail. I agree to these changes of adding two authors to the author lists of manuscript (DDISCOVERY-23-0180R1).

Zihui Ye

2023年7月5日 11:45，郭旭东 <19504@tongji.edu.cn> 写道：

Dear Zihui Ye,
This E-mail is asking to obtain your agreement about the additions of the following authors (Mr Jianguo Li and Mr Ke Feng) to the author lists of manuscript (DDISCOVERY-23-0180R1). And you might reply to this email and confirm that you are agree to these changes.

Best wishes,
Xudong Guo

----

(7) Agreement from Mr. Ke Feng

Dear Prof. Guo,
Thanks for your E-mail. I agree to these changes of adding two authors to the author lists of manuscript (DDISCOVERY-23-0180R1).

------------------ 原始邮件 ------------------

发件人: "郭旭东" <19504@tongji.edu.cn>;
发送时间: 2023 年 7 月 5 日(星期三) 中午 11:46
收件人: "枫叶蓝"<kefeng_bio@foxmail.com>;
主题: Asking to obtain the agreement about the author addition

Dear Mr. Ke Feng,
This E-mail is asking to obtain your agreement about the additions of the following authors (Mr Jianguo Li and Mr Ke Feng) to the author lists of manuscript (DDISCOVERY-23-0180R1). And you might reply to this email and confirm that you are agree to these changes.

Best wishes,
Xudong Guo

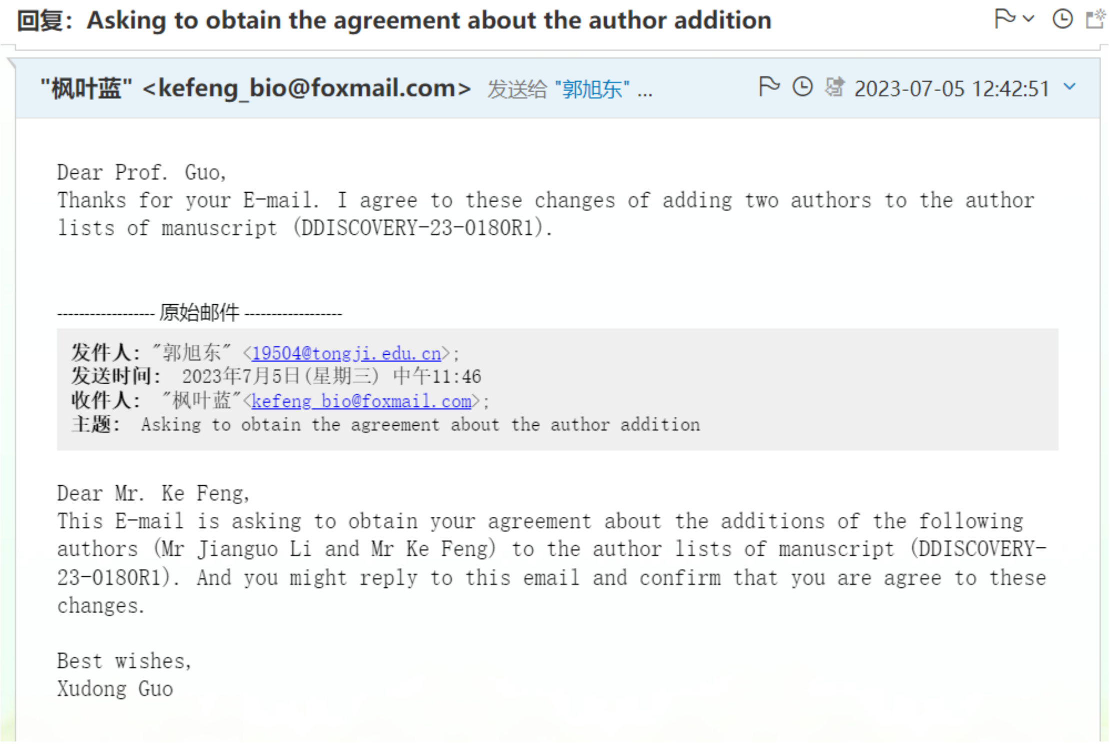

回复: Asking to obtain the agreement about the author addition

"枫叶蓝" <kefeng_bio@foxmail.com> 发送给 "郭旭东" ... 2023-07-05 12:42:51

Dear Prof. Guo,
Thanks for your E-mail. I agree to these changes of adding two authors to the author lists of manuscript (DDISCOVERY-23-0180R1).

------------------ 原始邮件 ------------------

发件人: "郭旭东" <19504@tongji.edu.cn>;
发送时间: 2023年7月5日(星期三) 中午11:46
收件人: "枫叶蓝"<kefeng_bio@foxmail.com>;
主题: Asking to obtain the agreement about the author addition

Dear Mr. Ke Feng,
This E-mail is asking to obtain your agreement about the additions of the following authors (Mr Jianguo Li and Mr Ke Feng) to the author lists of manuscript (DDISCOVERY-23-0180R1). And you might reply to this email and confirm that you are agree to these changes.

Best wishes,
Xudong Guo

(8) Agreement from Dr. Yiwei Yang

Dear Prof. Guo,

Thanks for your E-mail. I agree to these changes of adding two authors to the author lists of manuscript (DDISCOVERY-23-0180R1).

Best wishes,

Yiwei Yang

-------- 原始邮件 --------

发件人： 郭旭东 <19504@tongji.edu.cn>

日期： 2023 年 7 月 5 日周三 11:46

收件人： tinyslark@163.com

主 题： Asking to obtain the agreement about the author addition

Dear Dr. Yiwei Yang,

This E-mail is asking to obtain your agreement about the additions of the following authors (Mr Jianguo Li and Mr Ke Feng) to the author lists of manuscript (DDISCOVERY-23-0180R1). And you might reply to this email and confirm that you are agree to these changes.

Best wishes,

Xudong Guo

回复：Asking to obtain the agreement about the author addition

Dear Prof. Guo,

Thanks for your E-mail. I agree to these changes of adding two authors to the author lists of manuscript (DDISCOVERY-23-0180R1).

Best wishes,

Yiwei Yang

-------- 原始邮件 --------

发件人： 郭旭东 <19504@tongji.edu.cn>

日期： 2023年7月5日周三 11:46

收件人： tinyslark@163.com

主 题： Asking to obtain the agreement about the author addition

Dear Dr. Yiwei Yang,

This E-mail is asking to obtain your agreement about the additions of the following authors (Mr Jianguo Li and Mr Ke Feng) to the author lists of manuscript (DDISCOVERY-23-0180R1). And you might reply to this email and confirm that you are agree to these changes.

Best wishes,

Xudong Guo

(9) Agreement from Dr. Yanxin Xu

Hi Prof. Guo,

Thanks for your email. I agree to these change of adding two authors to the author list of manuscript (DDISCOVERY-23-0180R1).

Best,
Yanxin

From: 郭旭东
Date: 2023-07-04 22:47
To: xu53151977
Subject: Asking to obtain the agreement about the author addition

Dear Dr. Yanxin Xu,
This E-mail is asking to obtain your agreement about the additions of the following authors (Mr Jianguo Li and Mr Ke Feng) to the author lists of manuscript (DDISCOVERY-23-0180R1). And you might reply to this email and confirm that you are agree to these changes.

Best wishes,
Xudong Guo

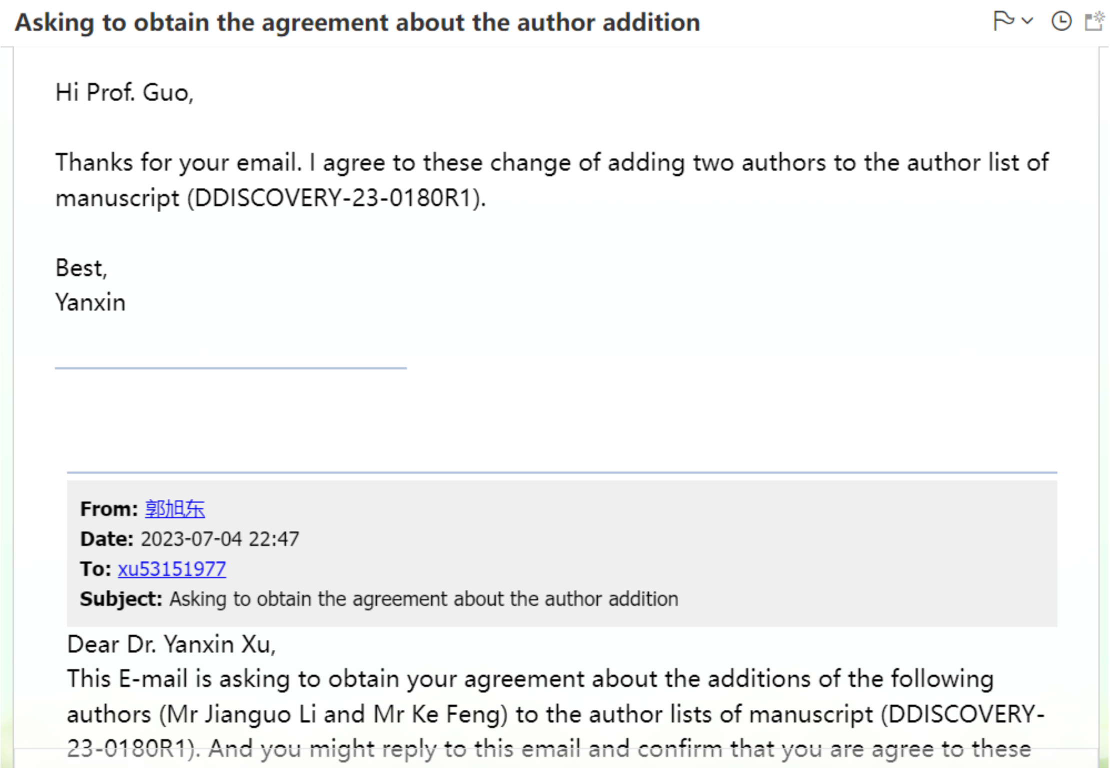
Asking to obtain the agreement about the author addition

Hi Prof. Guo,

Thanks for your email. I agree to these change of adding two authors to the author list of manuscript (DDISCOVERY-23-0180R1).

Best,
Yanxin

From: 郭旭东
Date: 2023-07-04 22:47
To: xu53151977
Subject: Asking to obtain the agreement about the author addition

Dear Dr. Yanxin Xu,
This E-mail is asking to obtain your agreement about the additions of the following authors (Mr Jianguo Li and Mr Ke Feng) to the author lists of manuscript (DDISCOVERY-23-0180R1). And you might reply to this email and confirm that you are agree to these

(10) Agreement from Prof. Jiuhong Kang

Dear Prof. Guo,
Thanks for your E-mail. I agree to the addition of two authors to the author lists of manuscript (DDISCOVERY-23-0180R1).

Jiuhong Kang

> -----原始邮件-----
> 发件人: "郭旭东" <19504@tongji.edu.cn>
> 发送时间: 2023-07-05 11:47:35 (星期三)
> 收件人: "康九红老师" <jhkang@tongji.edu.cn>
> 抄送:
> 主题: Asking to obtain the agreement about the author addition
>
> Dear Prof. Jiuhong Kang,
> This E-mail is asking to obtain your agreement about the additions of the following authors (Mr Jianguo Li and Mr Ke Feng) to the author lists of manuscript (DDISCOVERY-23-0180R1). And you might reply to this email and confirm that you are agree to these changes.
>
> Best wishes,
> Xudong Guo

Re: Asking to obtain the agreement about the author addition

Dear Prof. Guo,
Thanks for your E-mail. I agree to the addition of two authors to the author lists of manuscript (DDISCOVERY-23-0180R1).

Jiuhong Kang

> -----原始邮件-----
> 发件人: "郭旭东" <19504@tongji.edu.cn>
> 发送时间: 2023-07-05 11:47:35 (星期三)
> 收件人: "康九红老师" <jhkang@tongji.edu.cn>
> 抄送:
> 主题: Asking to obtain the agreement about the author addition
>
> Dear Prof. Jiuhong Kang,
> This E-mail is asking to obtain your agreement about the additions of the following authors (Mr Jianguo Li and Mr Ke Feng) to the author lists of manuscript (DDISCOVERY-23-0180R1). And you might reply to this email and confirm that you are agree to these changes.
>
> Best wishes,
> Xudong Guo
>
